# Uncovering the genetic basis of milk production traits in Mexican Holstein cattle based on individual markers and genomic windows

**DOI:** 10.1371/journal.pone.0314888

**Published:** 2025-02-03

**Authors:** José G. Cortes-Hernández, Adriana García-Ruiz, Francisco Peñagaricano, Hugo H. Montaldo, Felipe J. Ruiz-López

**Affiliations:** 1 PhD Program in Animal Health and Production Science, National Autonomous University of Mexico, Mexico, CDMX, Mexico; 2 National Center for Disciplinary Research in Animal Physiology and Improvement of the National Institute of Forestry, Agriculture and Livestock Research, Ajuchitlán, Querétaro, Mexico; 3 Department of Animal and Dairy Sciences, University of Wisconsin-Madison, Madison, Wisconsin, United States of America; 4 Department of Genetics and Biostatistics, Faculty of Veterinary Medicine and Husbandry, National Autonomous University of Mexico, Mexico, CDMX, Mexico; 5 Faculty of Higher Studies Cuautitlán, National Autonomous University of Mexico, Mexico, CDMX, Mexico; National Cheng Kung University, TAIWAN

## Abstract

The objective of this study was to evaluate the proportion of genetic variance explained by single nucleotide polymorphism markers, individually or clustered in 1, 2, and 5 Mb windows, for milk yield, fat yield, protein yield, fat content, protein content, and somatic cell score in Mexican Holstein cattle. The analysis included data from 640,746 lactation records of 358,857 cows born between 1979 and 2019, distributed in 353 herds in 18 states of Mexico. The analysis included genotypic data on 7,713 cows and 577 sires, with information on 88,911 markers previously imputed and filtered by quality control. Genomic scans via the single-step genomic best linear unbiased prediction method were performed using BLUPF90 software. A total of 162 markers were significantly associated (p<0.01) with the phenotypic traits evaluated, and the SNP markers were distributed across chromosomes 1, 3, 5, 6, 10, 12, 14, 16, 18, 20, 22, and 29. When the size of the genomic windows was increased from 1 to 5 Mb, a greater proportion of genetic variance was explained by the SNPs within the window, and a greater number of windows explained more than 1% of the genetic variance. The most significant regions were associated with two or more phenotypic traits, such as one region on chromosome 14 that harbors the DGAT1, EXOSC4, PPP1R16A, and FOXH1 genes, which affect all the traits under study. In general, the utilization of genomic windows resulted in a greater proportion of genetic variance explained by milk production traits.

## Introduction

Selection based on phenotypic and pedigree information has resulted in substantial improvements in genetic merit in different animal species [1]. Recent advances in genome sequencing and high-throughput genotyping technologies combined with genome-wide association studies (GWASs) have made it possible to identify single nucleotide polymorphisms (SNPs) associated with economically important traits in farm animals [2]. In cattle, as well as other animal species, such studies contribute to a better understanding of the genetic architecture of polygenic traits (Quantitative Trait Loci (QTLs)) such as production, reproduction, and health. Additionally, they may help improve the efficiency of selection processes [3].

For Holstein dairy cattle, GWASs have been carried out in populations in different parts of the world. In 2021, Bakhshalizadeh et al. [3] carried out a meta-analysis to identify SNPs associated with milk production traits. The meta-analysis included 26 studies carried out from 2010 to 2019 in countries such as Canada, China, France, Germany, Italy, Australia and Ireland. These authors reported that 1,583 SNPs were significantly associated with milk production traits (p<0.05). Additionally, the meta-analysis identified 9 QTLs for milk yield, 36 QTLs for fat percentage (fat content, FC), and 10 QTLs for protein percentage (protein content, PC). Some QTLs were confirmed on chromosome (BTA: *Bos taurus* autosome) 14, close to the DGAT1 gene associated with milk yield (MY), FC, and PC, and other SNP in BTA 14 close to the PPP1R16A gene associated with MY and FC. In the USA Holstein cattle population, four BTAs, namely, 5, 6, 14, and 20, had significant SNPs with effects on milk production traits. The 30.03–36.67 Mb region of BTA 20 harbors the GHR gene, which affects MY [4].

High-density SNP arrays are used in GWASs [4, 5], and the SNPs contained within a specific BTA segment tend to be correlated owing to their proximity and the large number of markers included in such arrays. GWAS conducted to test individual SNPs associated with genes has proven to be effective, but in some cases, there is more than one SNP associated with the same gene, or there are interactions between SNPs associated with different genes. One way to address this problem is to perform associations based on genomic windows, in which a larger number of SNPs with important effects are included simultaneously [6]. However, when arrays do not include SNPs across the whole genome, tests based on genomic windows can lead to the detection of spurious associations [7].

Although GWASs allows for the estimation of marker effects or the proportion of explained genetic variance (EGV) by genomic segments, the current lack of consensus in defining the optimal SNP window size has led to multiple alternative approaches [8]. In dairy cattle, Buaban et al. [9] utilized fixed windows of 1 Mb or 20 adjacent SNPs for the analysis of milk traits, whereas Zhou et al. [10] used five consecutive SNPs for the analysis of milk traits, and both considered windows explaining more than 0.5% of genetic variance to be significant. In studies carried out for other traits and breeds, the percentage of explained genetic variance (EGV) defined as important was different. For example, Li et al. [5] used windows of five adjacent SNPs (38.4 kb on average) in GWAS for residual feed intake in Holstein cattle. In Simmental cattle Zhuang et al. [11] used windows of 20 consecutive SNPs and defined windows explaining more than 1% of EGV for growth traits as significant. Zhuang et al. [11] concluded that windows explaining more than 1% of the EGV were usually >1 Mb [9].

The first GWAS for milk production traits in the Mexican Holstein population was carried out in 2014 and included 1,975 animals with information for 34,856 SNPs. Notably, none of the main genes affecting production traits, namely, DGAT1, ABCG2, or GHR, were found to be significant [12]. The study described herein is the first to consider a substantial number of genotyped animals and markers for the Mexican Holstein population. The findings of this study have the potential to enhance our comprehension of the selection process in this cattle population and shed light on candidate genes and gene networks associated with production traits.

The objective of this study was to evaluate the proportion of genetic variance explained by SNP markers either individually or clustered within 1, 2, and 5 Mb genomic windows for MY, FY, PY, FC, PC, and somatic cell score (SCS) in Mexican Holstein cattle and to identify candidate markers and candidate genes.

## Materials and methods

### Phenotypic and pedigree information

The database consists of 663,942 milk production records, excluding animals with an age at first calving of less than 16 months or older than 78 months at the third calving. In the end, the analysis considered 640,746 records from first (45.26%), second (33.72%) and third (21.02%) lactation, previously adjusted to 305 days and mature equivalent, corresponding to 358,857 Mexican Holstein cows born from 1979 to 2019, including information on MY, FY, PY in kilograms, FC, PC in percentage and SCS expressed on a linear scale of 0 to 9 (SCS = Log_2_(somatic cell count/1000,000) +3; [13]). The animals were distributed across 353 herds in 18 states of Mexico. The pedigree file contained 470,695 individuals with an average of five generations, 17,220 sires, and 161,757 dams. Phenotypic and pedigree information was provided by the Mexican Holstein Association.

### Genomic information

In this study, the following genotypes of animals from different densities were used: BovineLD v2.0 9K (4.15%), GGP Bovine LD v3.0 26K (5.91%), GGP Super LD v4.0 26K (1.38%), BovineSNP v3 50K (9.16%), GGP LD 77K (15.42%), GGP Bovine 100K (0.03%), GGP HD 150K (59.77%), Genome-Wide BOS 1 Bovine Array 640K (2.47%), and GGP HD 777K (1.71%) [14–16], which were previously imputed with the process described by García-Ruiz et al. [17] to 116,204 SNPs using FindHap V2 [18].

After quality control was applied to the imputed genotypes, 7,713 cows and 577 sires with information on 88,911 SNPs were included in this study. The genotyped sires had from 1 to 2,404 progeny in the pedigree file, but the number of genotyped daughters ranged from 1 to 133. The quality control for genotyped animals consisted of excluding those with call rates of <0.95, parent‒progeny Mendelian conflicts >1%, whereas the quality control of SNPs consisted of excluding those with a minor allele frequency of <0.05, call rate <0.95, or a p-value for Hardy-Weinberg equilibrium test <0.15. Quality control was carried out with the PreGSf90 program [19]. Genomic data were provided by the National Center for Disciplinary Research in Animal Physiology and Improvement of the National Institute for Forestry, Agriculture, and Livestock Research of Mexico.

### Prediction of breeding values

The estimated breeding values (EBVs) for the six traits were obtained via univariate single-step genomic best linear unbiased prediction (ssGBLUP) analysis [20–22] using BLUPF90 software [22]. The model used to obtain the EBV for each trait was as follows [23]:

$$y_{ijklmn}=\mu +{HYS}_{i}+{AGE}_{j}+PE_{k}+SH_{l}+ANIMAL_{m}+e_{ijklmn}$$ (1)

where *y*_*ijklmn*_ represents an observation (phenotype) for each of the analyzed traits and fixed effects were *μ* as the overall mean; *HYS*_*i*_ represents the i-th herd-year-season level (6,831 levels; 40 years and 2 seasons: January to June and July to December); and *AGE*_*j*_ represents the j-th age level at calving within lactation, with each lactation divided into three classes, thus, classified from 1 to to 9 for all lactations; within lactation, the levels were divided according to their distribution. The limits for the classes in months of age were as follows: 1 ≤ 23.4, 2 ≥ 23.5 and ≤ 25.5 and 3 ≥ 25.6 for first lactation; 4 ≤ 35.9, 5 ≥ 36 and ≤ 39.5 and 6 ≥ 39.6 for second lactation; and 7 ≤ 48.7, 8 ≥ 48.8 and ≤ 53.3 and 9 ≥ 53.4 for third lactation. This classification is based on the effect that age at calving has on the genetic evaluation of milk production traits [23]. The random effects included *PE* which is the k-th level of the permanent environment; *SH* which is the l-th level of the sire-herd combination (40,742 levels); *ANIMAL* which represents the animal effect and *e*_*ijklmn*_ which is the residual effect.

Because not all EBVs are estimated with the same accuracy due to differences in pedigree information or the availability of genotypic information and because these differences can influence the GWAS results, weights (1/PEV) for the animal observations were used in the analysis of all traits [24].

### GWAS analysis

Before the GWASs, a population structure analysis was performed by PLINK 1.9 [25] with the function—pca (principal components analysis) to detect the homogeneity of the population.

After EBVs prediction, a GWAS was performed by predicting the SNP marker effects based on the EBVs [ssGWAS proposed by Wang et al., 20]. The ssGWAS method is a modification of BLUP with the inverse of the numerator relationship matrix ***A***^−1^ replaced by the ***H***^−1^ matrix [26, 27] using the postGSf90 program [19]:

$$H^{-1}=A^{-1}+\left\lceil \begin{array}{cc} 0 & 0\\ 0 & G^{-1}-A_{22}^{-1} \end{array}\right\rceil$$ (2)

where $A_{22}^{-1}$ is the inverse numerator relationship matrix for genotyped animals and where ***G***^−1^ is the inverse of a genomic relationship matrix [28]:

G=ZDZ´/k (3)

where ***Z*** is the center matrix ***Z*** = ***M***−2*p*, where *M* is the maker allele count matrix, and where p is the allele frequency: ***D*** is a diagonal matrix with weights, and *k* is a scale parameter [28].

SNP effects ($\hat{a}$) were calculated via single-step genomic BLUP analysis [22] as follows:

$$\hat{a}=kDZ\prime G^{-1}\hat{u}$$ (4)

where k=12Σpi(1−pi), ***D*** is a diagonal matrix of weights representing the variances of the SNP as proposed by VanRaden [28], ***Z***′ is the SNP matrix adjusted by allele frequencies, ***G***^−1^ is the inverse matrix of genomic relationships and $\hat{u}$ is the EBV [21].

P-values were calculated as follows [8]:

$${p‐val}_{i}=2\left(1‐\Phi (\left|\frac{{\hat{a}}_{i}}{sd({\hat{a}}_{i})}\right|)\right)$$ (5)

where *p*-*val*_*i*_ is the p-value for each SNP, *Φ* is the cumulative standard normal function, ${\hat{a}}_{i}$ is the predicted effect of the i-th marker, and *sd* is the standard deviation of the i-th marker. The limit of significant association (p<0.01) was defined with the Bonferroni adjustment as -log10 (0.01/88,911) = 6.95 [8, 29].

### Calculation of the variance explained by individual SNPs and genomic windows

In the GWAS analysis, EGV was calculated by individual SNPs and genomic windows of different sizes (1, 2, or 5 Mb). The selection of window size was based on reports that concluded that 1 Mb to 5 Mb windows were appropriate for the analysis of complex traits (productive or reproductive) [9, 30, 31].

The EGV by one SNP is defined as:

$${EGV}_{SNP}=(2pq{{\hat{a}}_{i}}^{2})$$ (6)

where *p* is the frequency of the *A* allele and *q* is that of the *a* allele [32].

The EGV by window was calculated according to the methodology proposed by Wang et al. [25]:

$${EGV}_{w}=\frac{var(a_{i})}{EGV}x\;100=\frac{var(\Sigma_{j=1}^{n}z_{j}{\hat{u}}_{j})}{EGV}x100$$ (7)

where *a*_*i*_ is the genetic value of the i-th region of the n-adjacent SNP within the genomic window (1, 2 or 5 Mb), EGV is the genetic variance explained by all SNPs, *z*_*j*_ is the vector of the genetic content of the j-th SNP for all animals, and ${\hat{u}}_{j}$ is the marker effect of the j-th SNP within the i-th region.

The EGV by all SNPs was defined as described by Lourenco et al. [21] and Legarra et al. [32];

$$EGV=\sum_{i}^{SNP}\;{EGV}_{SNP}=2\sum_{i}^{snp}p_{i}\;q_{i}\;{{\hat{a}}_{i}}^{2}$$ (8)

Elements previously defined in Formulas (4) and (5).

Although there is no statistically established limit to define the percentage of genetic variance as significant, in this study, and according to Lourenco et al. [21] and Marques et al. [31], windows explaining more than 1% of the EGV were considered important because this limit, according to the length of the genome, which is ~3000 Mb, is 33 times the expected explained variance for each 1 Mb window, which was 0.03% (3000 Mb/100).

The SNPs found to be significant in the GWASs and important in the percentage of EGV were searched on the basis of their name and position on the QTLdb website https://www.animalgenome.org/cgi-bin/QTLdb/index [33] to identify the candidate genes or associated traits previously reported in other studies in this breed of cattle. Some interactions between genes previously reported and predicted via the STRING platform were also explored to understand the connections between genes [34].

To compare the contributions of individual SNPs and genomic windows in the GWAS analysis, we compared the EGV of the important windows with the sum of the EGV of individual SNPs located in the same genomic regions.

## Results

Descriptive statistics of the production traits and milk composition of the analyzed population of Holstein cattle from Mexico are shown in Table 1.

**Table 1:** Descriptive statistics of the analyzed traits.

Trait	Records	Mean	SD	Minimum	Maximum
MY (kg)	640,746	10991.42	22954.96	1500	22000
FY (kg)	204,873	393.81	96.68	47	938
PY (kg)	204,873	363.30	81.81	42	878
FC (%)	204,873	3.39	0.53	1.5	6.08
PC (%)	204,873	3.12	0.27	1.5	6.44
SCS	171,890	1.92	1.42	0.1	9

MY: Milk yield, FY: Fat yield, FC: Fat content, PC: Protein content, PY: Protein yield, SCS: Somatic cell score, SD: Standard deviation, kg: Kilograms, and %: Percentage.

**Fig 1:**
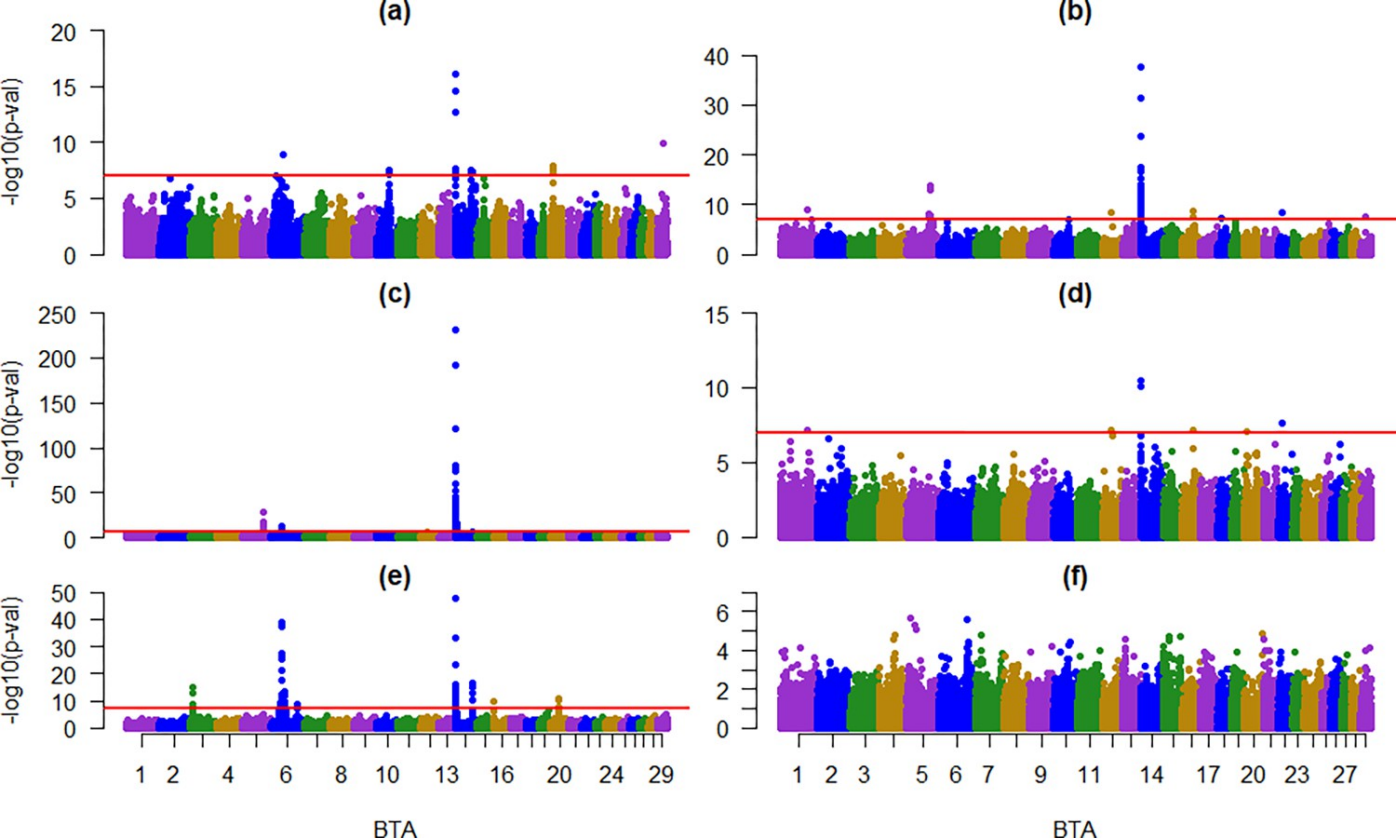
Manhattan plots for GWAS of the six traits studied in Mexican Holstein cattle. (a): Milk yield, (b): Fat yield, (c): Fat content, (d): Protein yield, (e): Protein content, and (f): Somatic cell score.

The results of the principal components analysis revealed a homogeneous structure of the analyzed population (SF1). A total of 23 statistically significant (p<0.01) SNPs were associated with MY, 49 with FY, 78 with FC, seven with PY, and 90 with PC. None were associated with the SCS (Fig 1).

Statistically significant (p<0.01) SNPs were detected in BTAs 1, 3, 5, 6, 10, 12, 14, 16, 18, 20, 22, and 29. S1 Table presents all the significant SNPs for all the traits and their associations with economically important traits, candidate genes, and QTLs previously reported [33]. In BTA 6, several markers associated with PC and FC were located within the ABCG2 gene region.

### Percentage of EGV by individual markers

In the analysis of individual SNPs, none explained more than 1% of the EGV. Only for the FC trait did some markers have an EGV close to 1%. The three markers associated with the maximum values of the -log10 (p-value) association for production traits and milk composition were ARS-BFGL-NGS-4939, ARS-BFGL-NGS-57820, and BovineHD1400000262 in BTA 14. The percentage of EGV for each of these markers is presented in Table 2.

**Table 2:** Percentage of EGV by individual markers with the greatest association according to GWASs.

SNP	BTA	MY	FY	FC	PY	PC	SCS
ARS-BFGL-NGS-4939	14	0.05	0.12	0.88	0.034	0.21	0.00004
ARS-BFGL-NGS-57820	14	0.05	0.11	0.85	0.037	0.17	0.00012
BovineHD1400000262	14	0.04	0.09	0.56	0.025	0.12	9.5E-08

SNP: Single nucleotide polymorphism, BTA: *Bos taurus* autosome, MY: Milk yield, FY: Fat yield, FC: Fat content, PC: Protein content, PY: Protein yield, SCS: Somatic cell score, EGV: SNP explained genetic variance.

### Percentage of EGV by genomic window size for all traits

Although some regions accounting for more than 1% of the EGV in the 1 Mb analysis were also observed within the 2 Mb and 5 Mb windows, distinct regions were also detected, and to a greater extent, by increasing the genomic window size in the analysis (S2–S7 Figs).

As may be observed in Table 3, increases in the size of the genomic window in Mb are positively associated with a higher proportion of EGV, a pattern observed in all traits, ranging from 1.32% to 7.07% for the SCS and a maximum of 27.55% for the FC.

**Table 3:** Percentage of EGV by genomic window size for all traits.

Traits	Window in Mb		
	1	2	5
MY	10.99	11.07	16.07
FY	4.79	8.05	12.17
FC	21.92	24.39	27.55
PY	3.40	3.95	8.38
PC	15.36	18.37	21.92
SCS	1.32	3.28	7.07

MY: Milk yield, FY: Fat yield, FC: Fat content, PC: Protein content, PY: Protein yield, SCS: Somatic cell score, Mb: Megabase, EGV: SNP explained genetic variance.

Increases in EGV were detected for some genomic regions when the analysis was conducted with 5 Mb genomic windows. For example, the SNP ARS-BFGL-NGS-57820, which had an EGV of 0.85 for FC in the analysis conducted with individual SNPs, increased to 18.65% when this SNP was located within the 5 Mb region. Similarly, the SNP ARS-BFGL-NGS-4939 marker changed from 0.88% to 15.46% (S4 Fig). Furthermore, increases were observed in the MY, although with a different order of magnitude. For example, the ARS-BFGL-NGS-4939 individual marker increased from 0.05 to 0.86% within 5 Mb windows.

For MY and FY, markers within 1, 2, and 5 Mb windows explaining more than 1% of the EGV on BTAs 3, 6, and 20 (S2, S3, and S5 Figs), markers for FC and PC on BTA 14 (S4 and S6 Figs) and markers for SCS in BTA 6 (S7 Fig) were observed.

Compared with the smaller window analysis, the results of the GWASs with the genomic window demonstrated that the 5 Mb analysis increased the number of significant windows and markers (EGV >1%), except for FC, in which four significant windows were located with 1 Mb analysis, three with 2 Mb analysis, and five with 5 Mb analysis.

Some windows were found to be significant for more than one trait, covering the same regions in all three window sizes (Table 4), but did not explain the same proportion of EGV for the associated traits. For example, the 5 Mb window (BovineHD1400000143 // BovineHD1400001709) on BTA 14 explained 1.02% for MY, 5.56% for PC, 6.04% for FY, and 22% for FC. Significantly smaller windows were also found in this region, such as BovineHD1400000785 // Hapmap23302-BTC-052123 and BovineHD1400000143 // BovineHD4100010534 in the 1 Mb analysis and ARS-BFGL-NGS-74378/ARS // BFGL-BAC-24839 and BovineHD1400000143 // BovineHD1400000629 in the 2 Mb analysis.

**Table 4:** Windows of different sizes (1, 2, and 5 Mb) explaining more than 1% of the EGV for more than one trait from MY, FY, FC, PY, PC, and the sum of EGV by individual markers located in the same region.

SNP START	SNP END	SNP	WMb		BTA	STR	END	TRA (EGV)	SNP-EGV
BovineHD1400000785	Hapmap23302-BTC-052123	55	1	14		3.85	4.85	FY (1.48), FC (2.56).	FY (0.46), FC (0.82).
BovineHD1400000143	BovineHD4100010534	29	1	14		0.24	1.20	FY (2.29), FC (14.92), PC (3.38).	FY (1.40), FC (8.84), PC (1.97).
BovineHD0600010463	Hapmap32210-BTC-035534	512	2	6		37.84	39.84	FC (1.94), PC (9.38).	FC (0.57), PC (1.96).
ARS-BFGL-NGS-74378	ARS-BFGL-BAC-24839	99	2	14		3.64	5.63	FY (2.29), FC (3.97).	FY (0.72), FC (1.30).
BovineHD1400000143	BovineHD1400000629	60	2	14		0.24	2.26	FY (3.28), FC (18.48), PC (4.65).	FY (1.91), FC (10.81), PC (2.57).
BovineHD2000001291	BovineHD2000001898	410	2	20		4.01	6.00	MY (5.21), PY (2.81).	MY (0.92), PY (0.53).
ARS-BFGL-NGS-79934	ARS-BFGL-NGS-68522	345	5	3		48.69	53.69	MY (2.13), PY (1.10).	MY (0.48), PY (0.25).
BovineHD0700017325	BovineHD0700019111	243	5	7		60.52	65.52	MY (1.27), PY (1.20).	MY (0.51), PY (0.38).
BovineHD1400000143	BovineHD1400001709	203	5	14		1.43	6.42	MY (1.02), FY (6.04), FC (22), PC (5.65).	MY (0.91), FY (2.73), FC (12.26), PC (2.97).

SNP: Number of single nucleotide polymorphism in the window, EGV: SNP explained genetic variance as percentage by windows analyses, WMb: window size in megabases, BTA: *Bos taurus* autosome, STR; Start position in megabases, END: End position in megabases, TRA: Trait, MY: Milk yield, FY: Fat yield, FC: Fat content, PC: Protein content, PY: Protein yield. SNP-EGV: Sum of SNP explained genetic variance as percentage by individual markers into the windows

In contrast with the percentage of EGV to the traits MY, FY, FC, PY, and PC by the different windows, the sum of EGV as a percentage to SNP inside this window in the analysis of individual markers was lower for all traits (last column of Table 4), and the sum of EGV to any of the individual markers into the windows did not reach 1% of EGV, as in the case of window analyses, for example, the window BovineHD1400000785 // Hapmap23302-BTC-052123 to FC (Table 4).

### Percentage of EGV by genomic window size for PY and MY

Some windows presented a positive association between their size in Mb and the EGV, except for the significant windows for PY and MY on BTA 3, where the 1 Mb window for BovineHD0300015741 // BovineHD0300016020 (51.93–52.93 Mb) of 200 SNPs explained 1.26% of the EGV for PY. This percentage decreased to 1.10% for the ARS-BFGL-NGS-79934 // ARS-BFGL-NGS-68522 5 Mb window (48.50–53.53 Mb) with 345 SNPs, covering the 1 Mb window (Table 5).

**Table 5:** Windows of different sizes (1, 2, and 5 Mb) explaining more than 1% of the EGV of PY.

SNP START	SNP END	SNP	EGV	WMb	BTA	STR	END
BovineHD0300015741	BovineHD0300016020	200	1.26	1	3	51.93	52.93
BovineHD2000001533	ARS-BFGL-NGS-5145	229	2.13	1	20	4.94	5.93
BovineHD0300015674	BovineHD0300016240	252	1.14	2	3	51.68	53.67
BovineHD2000001291	BovineHD2000001898	410	2.81	2	20	4.10	6.09
ARS-BFGL-NGS-79934	ARS-BFGL-NGS-68522	345	1.10	5	3	48.50	53.53
BovineHD0700017325	BovineHD0700019111	243	1.20	5	7	58.53	63.52
ARS-BFGL-NGS-10149	BovineHD0900031176	270	1.10	5	9	99.17	104.10
BovineHD1700000602	BTB-01310918	248	1.13	5	17	2.72	7.71
BovineHD2000000327	ARS-BFGL-NGS-26474	537	2.22	5	20	1.14	6.12
BovineHD2000011112	BovineHD2000012563	202	1.61	5	20	39.08	44.09

SNP: Number of Single nucleotide polymorphism in the window, EGV: SNP explained genetic variance as percentage, WMb: Window size in megabases, BTA: *Bos taurus* autosome, STR; Start position in megabases, END: End position in megabases.

For MY, four statistically significant windows were found with the 1 Mb analysis, four with 2 Mb, and 10 with 5 Mb. The 1 Mb window on BTA 3, BTA-07805-no-rs // BovineHD0300016035, containing 198 SNPs, explained 3.10% of the EGV, and this value decreased to 2.13% for the 5 Mb window, which covered the previous window and contained more markers (SNP ARS-BFGL-NGS-79934 // ARS-BFGL-NGS-68522 with 345 SNPs). The same trend was observed for windows reported on BTA 6, where the 1 Mb window (BovineHD0600010587 // BovineHD0600010801) explained 2.31%, and the corresponding 5 Mb window explained 1.88% (Table 6).

**Table 6:** Windows of different sizes (1, 2, and 5 Mb) explaining more than 1% of the EGV of MY.

SNP START	SNP END	SNP	EGV	WMb	BTA	STR	END
BTA-07805-no-rs	BovineHD0300016035	198	3.10	1	3	52.01	52.99
BovineHD0600010587	BovineHD0600010801	228	2.31	1	6	36.80	37.79
BovineHD0700000034	BovineHD0700000286	139	1.10	1	7	0.31	1.26
MS-rs378237043	BovineHD2000001736	206	4.48	1	20	4.62	5.70
BovineHD0300015635	BovineHD0300016180	251	2.52	2	3	51.55	53.53
BovineHD0600010502	BovineHD0600010934	490	2.25	2	6	36.49	38.47
BovineHD1400006962	BovineHD1400007504	197	1.09	2	14	22.34	24.30
BovineHD2000001291	BovineHD2000001898	410	5.21	2	20	4.10	6.09
BovineHD0200012258	ARS-BFGL-BAC-47330	231	1.20	5	2	42.24	47.24
ARS-BFGL-NGS-79934	ARS-BFGL-NGS-68522	345	2.13	5	3	48.50	53.53
BovineHD4100004467	BovineHD0600011396	815	1.88	5	6	35.62	40.58
BovineHD0700017325	BovineHD0700019111	243	1.27	5	7	58.53	63.52
BovineHD0700000001	Hapmap57767-ss46527024	320	1.01	5	7	0.15	5.07
BovineHD1000016695	BovineHD1000017736	183	1.32	5	10	56.11	60.96
BovineHD1300013426	ARS-BFGL-BAC-15732	168	1.11	5	13	45.62	50.49
BovineHD1400000143	BovineHD1400001709	203	1.02	5	14	1.43	6.42
BovineHD2000000353	BovineHD2000001946	536	4.05	5	20	1.28	6.24
BTB-01189862	BovineHD2000012344	213	1.08	5	20	38.20	43.16

SNP: Number of Single nucleotide polymorphism in the window, EGV: SNP explained genetic variance as percentage, WMb: Window size in megabases, BTA: *Bos taurus* autosome, STR; Start position in megabases, END: End position in megabases, MY: Milk Yield.

### Percentage of EGV by genomic window size for FY and FC

For FY, three significant windows were found in the 1 Mb analysis, increasing to four with 2 Mb and six with 5 Mb. In the GWAS of 5 Mb, three new windows located on BTAs 1, 6 and 10 were associated with this trait and in regions not reported in the analyses of 1 and 2 Mb. The two windows found on BTA 14 with the 1 Mb and 2 Mb analysis were located within a region of the single window reported on BTA 14 with the 5 Mb analysis (Table 7).

**Table 7:** Windows of different sizes (1, 2, and 5 Mb) explaining more than 1% of the EGV of FY.

SNP START	SNP END	SNP	EGV	WMb	BTA	STR	END
BovineHD1400000143	BovineHD4100010534	29	2.29	1	14	0.24	1.20
BovineHD1400000788	Hapmap23302-BTC-052123	54	1.48	1	14	2.84	3.82
BovineHD2000001529	BovineHD2000001834	231	1.02	1	20	4.92	5.92
BovineHD0300015681	Hapmap57193-rs29021598	252	1.05	2	3	51.70	53.68
ARS-BFGL-NGS-74378	ARS-BFGL-BAC-24839	99	2.29	2	14	2.62	4.59
BovineHD1400000143	BovineHD1400000629	60	3.28	2	14	0.24	2.26
BovineHD2000001292	BovineHD2000001898	409	1.43	2	20	4.11	6.09
BovineHD0100018022	BovineHD0100019406	213	1.03	5	1	63.04	68.04
BovineHD0300014791	BovineHD0300016180	345	1.12	5	3	48.52	53.53
ARS-USDA-AGIL-chr6-20764777-000720	BovineHD0600007100	210	1.02	5	6	19.49	24.38
BTA-88344-no-rs	BovineHD1000017665	196	1.44	5	10	55.64	60.50
BovineHD1400000143	BovineHD1400001709	203	6.04	5	14	1.43	6.42
BovineHD2000000234	BovineHD2000001818	520	1.52	5	20	0.90	5.89

SNP: Number of Single nucleotide polymorphism in the window, EGV: SNP explained genetic variance as percentage, WMB: Window size in megabases, BTA: *Bos taurus* autosome, STR; Start position in megabases, END: End position in megabases, FY: Fat yield.

In this study, three windows located on BTA 14 had the highest level of association with FC, all with the same start SNP but different sizes: BovineHD1400000143 // BovineHD4100010534 in 1 Mb with 29 SNPs, which explained 14.92% of the EGV, BovineHD1400000143 // BovineHD1400000629 in 2 Mb with 60 SNPs and 18.48% of the EGV, and BovineHD1400000143 // BovineHD1400001709 in 5 Mb, with the highest number of SNPs (203) and EGV (22%). This 5 Mb window covered the region of the 1 Mb and 2 Mb windows, and an additional window was found on BTA 20 in our study, a region not reported in smaller windows (Table 8).

**Table 8:** Windows of different sizes (1, 2, and 5 Mb) explaining more than 1% of the EGV of FC.

SNP START	SNP END	SNP	EGV	WMb	BTA	STR	END
BovineHD0600010465	BovineHD4100004579	271	1.71	1	6	36.42	37.41
Hapmap30374-BTC-002159	BovineHD1400000654	33	2.72	1	14	1.27	2.31
BovineHD1400000143	BovineHD4100010534	29	14.92	1	14	0.24	1.20
BovineHD1400000785	Hapmap23302-BTC-052123	55	2.56	1	14	2.83	3.82
BovineHD0600010463	Hapmap32210-BTC-035534	512	1.94	2	6	36.41	38.40
ARS-BFGL-NGS-74378	ARS-BFGL-BAC-24839	99	3.97	2	14	2.62	4.59
BovineHD1400000143	BovineHD1400000629	60	18.48	2	14	0.24	2.26
BTA-15560-no-rs	ARS-BFGL-NGS-15498	157	1.38	5	5	91.68	96.67
BovineHD0600009721	BovineHD4100004685	783	2.13	5	6	33.51	38.50
Hapmap33415-BTC-007589	ARS-BFGL-NGS-104239	202	1.04	5	14	8.58	13.36
BovineHD1400000143	BovineHD1400001709	203	22.00	5	14	1.43	6.42
UA-IFASA-9183	BovineHD2000010375	149	1.00	5	20	31.18	36.17

SNP: Number of Single nucleotide polymorphism in the window, EGV: SNP explained genetic variance as percentage, WMb: Window size in megabases, BTA: *Bos taurus* autosome, STR; Start position in megabases, END: End position in megabases, FC: Fat content.

### Percentage of EGV by genomic window size for PC and SCS

For PC, although the window was located on BTA 20, BTA-103550-no- rs // ARS-BFGL-NGS-97470, with 56 SNPs, explained 1.26%, whereas BovineHD2000010736 // BovineHD2000012211 in the same BTA but in a different region explained 1.03%, with 218 SNPs for PC. Notably, in our PC analysis, the three significant windows found with 1 Mb on BTA 6 were observed within a single window in the 5 Mb analysis, whereas another window was reported in this same BTA near this region (Table 9).

**Table 9:** Windows of different sizes (1, 2, and 5 Mb) explaining more than 1% of the EGV of the PC.

SNP START	SNP END	SNP	EGV	WMb	BTA	STR	END
BovineHD0600010492	BovineHD0600010759	258	8.62	1	6	36.47	37.46
BovineHD4100004585	BovineHD0600010936	240	1.14	1	6	37.50	38.49
BovineHD0600010202	BovineHD4100004511	141	1.19	1	6	35.19	36.20
BovineHD1400024152	BovineHD1400018736	42	1.03	1	14	63.85	64.81
BovineHD1400000143	BovineHD4100010534	29	3.38	1	14	0.24	1.20
Hapmap27887-BTA-156628	BovineHD4100004482	157	1.12	2	6	34.02	36.03
BovineHD0600010463	Hapmap32210-BTC-035534	512	9.83	2	6	36.41	38.40
BovineHD1400000143	BovineHD1400000629	60	4.65	2	14	0.24	2.26
BovineHD1400018374	BovineHD1400018897	70	1.50	2	14	63.52	65.49
BTA-103550-no-rs	ARS-BFGL-NGS-97470	56	1.26	2	20	31.03	33.03
BovineHD0600022987	BovineHD0600024251	203	1.26	5	6	81.91	86.89
BovineHD0600009722	BovineHD4100004685	782	9.40	5	6	33.52	38.50
BovineHD1400000143	BovineHD1400001709	203	5.65	5	14	1.43	6.42
BovineHD1400017653	BovineHD1400019188	175	2.47	5	14	61.44	66.33
BovineHD2000010736	BovineHD2000012211	218	1.03	5	20	37.51	42.47
BovineHD2000008743	Hapmap57531-rs29013890	156	2.12	5	20	29.82	34.80

SNP: Number of Single nucleotide polymorphism in the window, EGV: SNP explained genetic variance as percentage, WMb: Window size in megabases, BTA: *Bos taurus* autosome, STR; Start position in megabases, END: End position in megabases, PC: Protein content.

For the SCS, a significant region was detected with all three window sizes. The 1 Mb window, which is within a 2 Mb window and both within the region analyzed within 5 Mb, has the highest EGV (2.87%), with the 2 Mb window and 1 Mb window explaining 2.26% and 1.32%, respectively (Table 10).

**Table 10:** Genomic windows explaining more than 1% of the SCS for different GWAS sizes (1, 2, and 5 Mb).

SNP START	SNP END	SNP	EGV	WMb	BTA	STR	END
BovineHD0600010642	BovineHD0600010842	225	1.32	1	6	37.02	38.00
BovineHD0600010486	BovineHD0600010926	500	2.26	2	6	36.46	38.44
BovineHD1500000259	BovineHD1500000701	82	1.01	2	15	0.88	2.85
BovineHD0600009809	BovineHD0600010988	796	2.87	5	6	33.79	38.74
BovineHD0600023080	BovineHD0600024368	212	1.15	5	6	82.26	87.22
BovineHD1300000057	BovineHD1300001379	201	1.00	5	13	0.51	5.36
BovineHD1400005502	BovineHD1400007036	354	1.05	5	14	17.66	22.61
BovineHD2000019311	BovineHD2000021003	239	1.00	5	20	66.77	71.76

SNP: Number of Single nucleotide polymorphism in the window, EGV: SNP explained genetic variance as percentage, WMb: Window size in megabases, BTA: *Bos taurus* autosome, STR; Start position in megabases, END: End position in megabases, SCS: Somatic cell score.

## Discussion

### Markers associated with milk production and composition traits

Among the 162 SNP markers associated with the studied traits, 37 have not been previously reported or associated with genes or traits of interest [33]. Neighbor associations have been reported in up to 10,000 base pairs to the right or left of the referenced position of some significantly associated markers in this study. On BTA 6, two SNPs (BovineHD0600010605 and BovineHD0600010435) were associated with PC, and two other SNPs (BovineHD0600010555 and BovineHD0600010569) were associated with FC and PC; furthermore, these SNPs were related to the ABCG2 gene, according to the QTLdb website [33] (S1 Table). This gene has been reported to be associated with saturated and unsaturated fatty acids in milk Holstein cattle [35]. In studies previously carried out in Holstein cattle from Mexico [36], runs of homozygosity of up to 7.56 Mb on BTA 10 have been reported, with positive effects on MY (+562.53 kg), FY (18.26 kg), and PY (14.63 kg). In this study, only three SNPs associated with MY were found in BTA 10, with different positions where the homozygosity runs were reported. For SCS, no associated SNPs were reported in this study, but in other studies in Mexican Holstein cattle, Duran et al. [37] reported that 24 and 47 copy number variation regions in BTAs 2, 7, 10, 13, 18, 19, 23, 28 and 29 were significantly associated with SCS through two different methods and identified 18 candidate genes that are part of two gene networks related to stress, cell death, inflammation, and the immune response.

In terms of the number of SNPs significantly associated with the traits examined here, important differences may be noted between our investigation and other studies. For example, Kim et al. [38] reported 104 significant markers for MY in Korean Holstein cattle, whereas 23 were observed in our analysis. Although both studies used imputed genotypes, the total number of markers included was also greater, with 201,704 and 88,911 SNP markers, respectively. In both cases, the most significant marker for MY was the diacylglycerol acyltransferase (DGAT1) gene (ARS-BFGL-NGS-4939).

In a meta-analysis conducted by Bakhshalizadeh et al. [3], a total of 1,583 SNPs were found to be significantly associated (p<0.05) with MY and milk components in Holstein cattle from Australia, Canada, China, France, Germany, Ireland, and Italy. Furthermore, several genes on BTA 14, such as DGAT1 and PPP1R16A, presented the greatest associations in most of the populations analyzed, which coincides with the findings of our study.

The DGAT1 gene, associated with the SNPs ARS-BFGL-NGS-4939, rs109421300 [39], and AX-311625843 [38], has the highest level of association with MY, FY, FC, PY, and PC reported in the literature [3]. This gene encodes acylcoenzyme A: diacylglycerol acyltransferase, a protein involved in fat metabolism, which displays polymorphisms resulting from the substitution of lysine for alanine at position 232 (K232A mutation). Specifically, the lysine variant (KK or BB genotype) has been associated with increased levels of FY and FC, whereas the alanine variant (AA genotype) has been associated with increased MY and PY in cattle [40].

Bakhshalizadeh et al. [3] reported that the SNP rs109146371, located on BTA 14 at position 1651311 bp, had the most significant association with MY. This SNP is associated with the PPP1R16A (protein phosphatase 1 regulatory subunit 16A) gene, which is responsible for protein encoding. In our population, this gene was found to be associated with the SNP ARS-BFGL-NGS-57820 and was significantly associated with MY, FY, PY, FC, and PC. Additionally, this gene has positive effects on MY and PC and negative effects on FY and FC [29].

### EGV by genomic windows and related genes

A total of 19 significant genomic windows were identified by 1 Mb analysis: 20 in 2 Mb and 38 in 5 Mb for the different traits examined. A study conducted in a Brazilian Holstein population [2] revealed that 46 genomic windows of 10 SNPs distributed on BTAs 1, 5, 6, 8, 9, 11, 12, 14, 16, and 19 explain more than 1% of the EGV for MY and its components, concurring with the results of our study on BTAs 5, 6, 9, and 14 and diverging in windows reported for BTAs 8, 11, 12, 16, and 19. Although a window related to SCS in BTA 8 was reported in the Brazilian population [2], other windows that were associated with traits not taken into account in our study, such as monounsaturated and polyunsaturated fatty acids and stearic acid, were identified. In addition, we observed windows in other BTAs, such as 2, 3, 7, 10, 13, and 17, that were associated with milk production traits. These discrepancies are probably due to differences in the size of the genomic window used for the analysis and the traits included in the studies.

**Fig 2:**
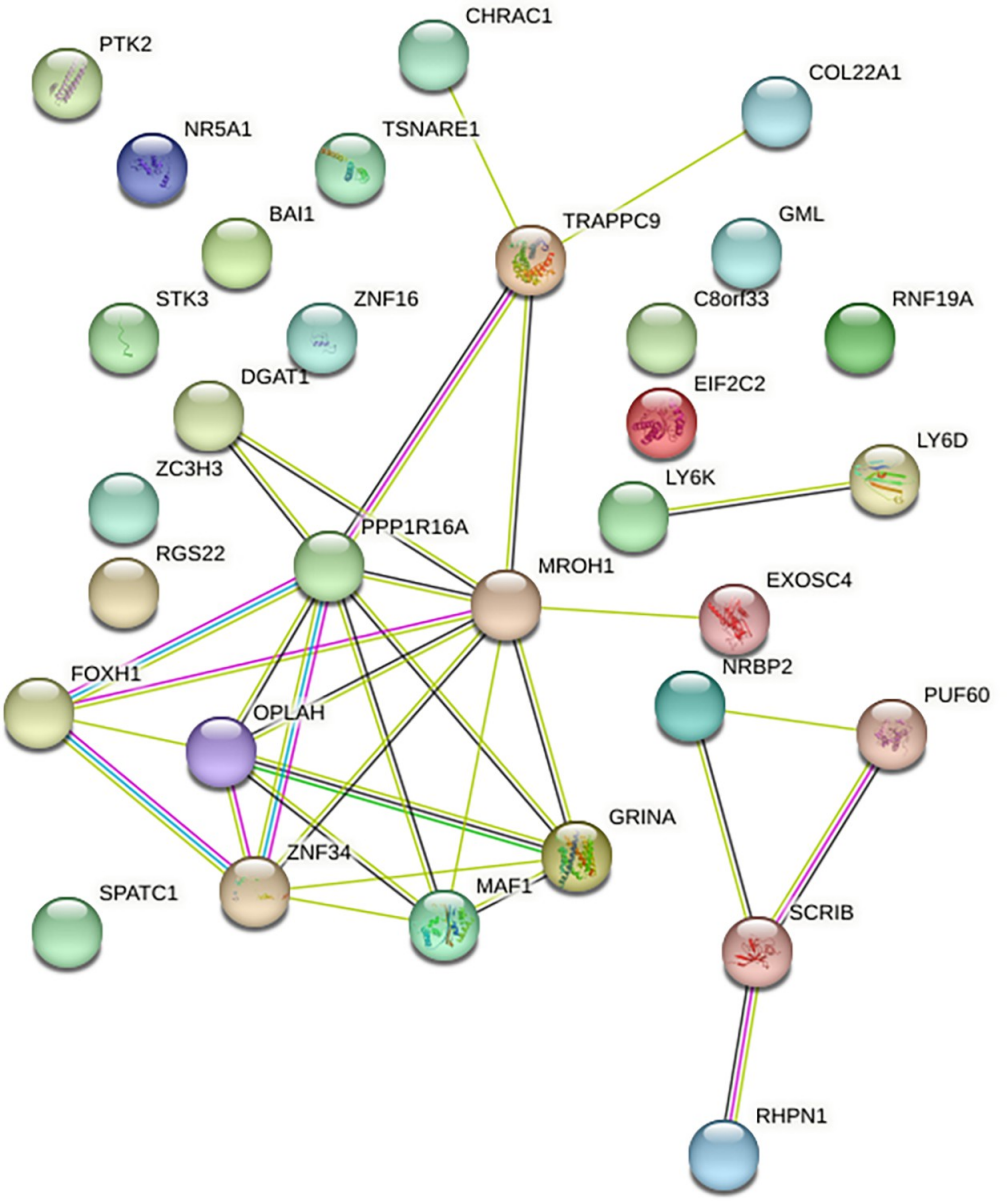
Interactions between genes associated with the significant SNPs on BTA 14 reported in the GWAS.

The genes DGAT1, PPP1R16A, EXOSC4 (Exome component 4; responsible for protein coding), FOXH1 (FAST1), MROH1 (HEATR7A) and OPLAH (5-OPase; ATP hydrolyzer) located on BTA 14 were identified within the window BovineHD1400000143 // BovineHD4100010534 and were also significantly associated (p<0.01) with the traits of MY, FY, FC, PY and PC studied here (S1 Table). There are interactions between some of these genes within this window on BTA 14, which can be observed in Fig 2, made with STRING-db.org; [34]. The interactions between the ZNF34, GRINA, MAF1, TRAPPC9, CHRAC1, and COL22A1 genes, as well as other isolated genes and other interactions, such as the NRBP2, PUF60, SCRIB, and RHPN1 genes and the LY6K and LY6D interaction, are illustrated in Fig 2. In the location of this interaction of genes from ZNF34 at position 0.31 Mb and up to the COL22A1 gene located at position 4.06 Mb, we observed a 2 Mb window (BovineHD1400000143 // BovineHD1400000629) explaining 18.48% of the EGV for FC and 3.28% for FY. This window also covers the 1 Mb window (BovineHD1400000143 // BovineHD4100010534), explaining 14.92% of the EGV for FC and 2.29% for FY. In turn, in the analysis with 5 Mb windows, a window detected in the position from 1.43 to 6.42 Mb (BovineHD1400000143 // BovineHD1400001709) explained up to 22% of the EGV for FC and 6.04% for FY, in addition to being associated with MY, PY and PC. These results suggest that a greater window size covers a greater number of genes, increasing the effect of markers due to adjacent SNPs interactions between them. This explains why the analysis of different window sizes increases the EGV for the markers in contrast with the analyses of individual SNPs (Table 4).

The 5 Mb window (BovineHD1400000143 // BovineHD1400001709) on BTA 14 was particularly notable, containing 29 of the 73 significantly associated SNPs (-log10(p) >6.95) reported in the analyses of the traits and the highest EGV for FC (22%). Atashi et al. [41], reported three windows (1.48–1.68, 1.85–2.11, and 2.67–2.94 Mb) within this region associated with MY at 305 days in Holstein cattle from Belgium, the Netherlands, Great Britain, and Denmark, but only one window (located between 1.48–1.68 Mb) explained more than 1% of the EGV (1.21%) for MY, possibly because of the small window size established for their analysis (50 SNPs) and because the authors were unable to identify more windows. Jiang et al. [4] also reported a window in a study conducted with 60,671 SNPs in U.S. Holstein cattle at the 1.42–5.49 Mb position, with great effects on MY and FY due to the DGAT1 gene. Ariyarathne et al. [42] observed a window at the position of 1 to 2 Mb in the same BTA with 22 SNPs that explained 38.2% of the EGV for FC traits in a study conducted with 45,062 markers in Holstein Friesian, Jersey, and crossbred cows. In Portuguese Holstein cattle close to the same region of BTA 14 (1.87–2.05 Mb), 3 SNPs were reported to have captured 2.7% of the EGV for MY in the 1st lactation without identifying the DGAT1 gene linked to one of these three SNPs [43]. Therefore, it may be surmised that a greater gene effect can be captured for some traits by increasing the size of the genomic window when there are genes with greater effects in the region, as in the case of the windows reported for FC (Table 7). Additionally, the difference may be explained by the differences in the number of markers used, the number of breeds, and the inclusion of crossbreeds. In our population, only one breed was included, and the population structure was found to be homogeneous after PLINK analysis.

In most windows, the greater EGV obtained was in the greater size (5 Mb, Table 4). For example, the genomic windows that explained up to 22% of the EGV for FC are possibly due to the presence of genes within the windows with greater effects on the trait combined with the high presence of SNPs in that region with linkage disequilibrium or interactions that are associated with the gene with major effects, such as DGAT1. Although in some cases, smaller windows individually showed higher levels of EGV, this occurred only in the BTA 3 and 6 windows for PY and MY (Tables 4 and 5). This may be because larger windows may include more SNP markers in the window with contributions close to zero to the breeding value (small effects) [30]. Other likely explanations are possible epistasis effects within BTA [44] or the location of some genes and the level of linkage disequilibrium between SNPs, being as this decreases as the distance between markers increases [45]. A study of dairy cattle from the Netherlands revealed that on BTA 6 in the region of 44.18 Mb to 88.53 Mb, several SNPs were significantly associated with PY, PC, β-casein, and κ-casein; two SNPs were the most significant, ULGR_BTC-060550, which is known to be in linkage disequilibrium with the polymorphisms responsible for protein variants A1 and A2 of β-casein and explained approximately 95% of the additive genetic variance to β-casein [46]. No associated SNPs were reported in the GWASs of MY and PY in our analysis (Tables 5 and 6) for the ARS-BFGL-NGS-79934 // ARS-BFGL-NGS-68522 window on BTA 3, which is in the region from 48.50 to 53.53 Mb. Therefore, better detection of regions influencing the EGV for the different traits examined in this study was usually achieved with 5 Mb windows.

The use of genomic windows in the GWAS improved the understanding of the genetic architecture of the traits evaluated, demonstrating that the percentage of EGV by SNPs for the traits increases because the SNPs are associated with genes with greater effects, and these genes are in DL with other genes with minor effects and because of the existence of interactions between them. This is important for the genetic evaluation of complex traits with low heritability that are difficult to measure or determined by polygenic effects, as in the case of SCS [47]. A greater proportion of the EGV of traits of economic importance can improve the estimate of heritability and increase the rate of genetic gain [48].

## Limitations

The observed interactions between genes determined via the STRING platform did not delve into the biological processes that influence the expression of the traits, which could elucidate the mechanistic pathways that may support the genetic effects observed in this study. Therefore, studies focused on analyzing the discovered genetic networks should be carried out.

The EGV is limited by the number of SNPs included in the analysis, and the use of long genomic windows could result in the inclusion of SNPs with no effect on the trait. This could cause bias in the estimation of the EGV. Because there is no consensus on defining the percentage of EGV as significant, many regions in the genome that have small contributions to complex traits are ignored.

## Conclusion

In both GWAS analyses, which utilized individual SNPs or windows, the regions previously associated with the studied traits were confirmed, and the results of some genes with major effects revealed differences in the EGV. Some regions in the genome with smaller windows (1 or 2 Mb) presented greater EGV, probably due to the major effects of certain genes. Conversely, larger windows (5 Mb) encapsulated more genetic effects linked to adjacent SNPs. Therefore, the use of genomic windows could improve the proportion of EGV for milk production traits since genomic windows could cover interactions between genes with effects on traits that are not captured by analyses of individual SNPs. This could benefit the estimation of the heritability of milk production traits and consequently improve genetic gain in populations.

## Supporting information

S1 Table SNPs associated with the traits of milk yield, fat yield and content, protein yield and content and somatic cell score. ALB: Blood albumin level, UH: Udder height, SNPn: SNP name, BH: β-Hydroxybutyrate content in milk, BTA: *Bos taurus* autosome, BTW: Birth weight, BW: Body weight, CA: Caprylic acid content in milk, CAL: Calcium content in milk, CAS: Casein content in milk, CE: Calving ease, CO: Milk cholesterol content, CR: Conception rate, CSW: Carcass weight, DMI: Dry matter intake, EV: Ejaculate volume, FC: Fat content, FE: Milk iron content, FX: Fertility index, FY: Fat yield, HI: Height, HT: Heat tolerance, KP: Milk kappa casein content, KT: Ketosis, LAC: Milk lactose production, LI: Linoleic acid content in milk, LIN: Linolenic acid content in milk, LM: Lean meat yield, LON: Longevity, LPL: Length of productive life, MB: Position in Mb, MI: Myristic acid content in milk, MS: Milking speed, MY: Milk yield, NM: Net merit, SB: Stillbirth, OL: Oleic acid content in milk, PAL: Palmitic acid content in milk, PAT: Palmitoleic acid content in milk, PC: Protein content, PLAC: Persistence of lactation, PY: Protein yield, P-FC: p-value for association of fat content, P-FY: p-value for association of fat yield, P-MY: p-value for association of milk yield, P-PC: p-value for association of protein content, P-PY: p-value for association of protein yield, QTL: Quantitative trait locus, RVL: Rear view of hind legs, RIV: Riboflavin content in milk, SCS: Somatic cell score, TP: Teat placement, TS: Tuberculosis susceptibility, UCF: Udder conformation, UC: Udder cleft. **ASSOCIATIONS, GENES, and QTLs** were searched on the QTLdb website https://www.animalgenome.org/cgi-bin/QTLdb/index [34] with the SNP location used as a reference.
(DOCX)

S1 Fig Genetic structure of the analyzed population. PC1: First principal component, PC2: Second principal component.
(TIF)

S2 Fig Manhattan plot for the EGV in each window size analysis (1 SNP, 1 Mb, 2 Mb, and 5 Mb) for MY. Mb: Megabase, BTA: *Bos taurus* autosome, MY: Milk yield, SNP: Single nucleotide polymorphism, % EGV: Explained genetic variance as a percentage.
(TIF)

S3 Fig Manhattan plot for the EGV in each window size analysis (1 SNP, 1 Mb, 2 Mb, and 5 Mb) for FY. Mb: Megabase, BTA: *Bos taurus* autosome, FY: Fat yield, SNP: Single nucleotide polymorphism, % EGV: Explained genetic variance as percentage.
(TIF)

S4 Fig Manhattan plot for the EGV in each window size analysis (1 SNP, 1 Mb, 2 Mb, and 5 Mb) for FC. Mb: Megabase, BTA: *Bos taurus* autosome, FC: Fat content, SNP: Single nucleotide polymorphism, % EGV: Explained genetic variance as percentage.
(TIF)

S5 Fig Manhattan plot for the EGV in each window size analysis (1 SNP, 1 Mb, 2 Mb, and 5 Mb) for PY. Mb: Megabase, BTA: *Bos taurus* autosome, PY: Protein yield, SNP: Single nucleotide polymorphism, % EGV: Explained genetic variance as percentage.
(TIF)

S6 Fig Manhattan plot for the EGV in each window size analysis (1 SNP, 1 Mb, 2 Mb, and 5 Mb) for PC. Mb: Megabase, BTA: *Bos taurus* autosome, PC: Protein content, SNP: Single nucleotide polymorphism, % EGV: Explained genetic variance as percentage.
(TIF)

S7 Fig Manhattan plot for the EGV in each window size analysis (1 SNP, 1 Mb, 2 Mb, and 5 Mb) for SCS. Mb: Megabase, BTA: *Bos taurus* autosome, SCS: Somatic cell score, SNP: Single nucleotide polymorphism, % EGV: Explained genetic variance as percentage.
(TIF)

## References

[1] Lu S, Liu Y, Yu X, Li Y, Yang Y, Wei M, et al. Prediction of genomic breeding values based on pre-selected SNPs using ssGBLUP, WssGBLUP and BayesB for Edwardsiellosis resistance in Japanese flounder. Genet Sel Evol. 2020 Dec;52(1):49. doi: 10.1186/s12711-020-00566-2 32811444 PMC7437005

[2] Iung LHS, Petrini J, Ramírez-Díaz J, Salvian M, Rovadoscki GA, Pilonetto F, et al. Genome-wide association study for milk production traits in a Brazilian Holstein population. J Dairy Sci. 2019 Jun;102(6):5305–14. doi: 10.3168/jds.2018-14811 30904307

[3] Bakhshalizadeh S, Zerehdaran S, Javadmanesh A. Meta-analysis of genome-wide association studies and gene networks analysis for milk production traits in Holstein cows. Livest Sci. 2021 Aug;250:104605.

[4] Jiang J, Ma L, Prakapenka D, VanRaden PM, Cole JB, Da Y. A Large-Scale Genome-Wide Association Study in U.S. Holstein Cattle. Front Genet. 2019 May 14;10:412. doi: 10.3389/fgene.2019.00412 31139206 PMC6527781

[5] Li B, Fang L, Null DJ, Hutchison JL, Connor EE, VanRaden PM, et al. High-density genome-wide association study for residual feed intake in Holstein dairy cattle. J Dairy Sci. 2019 Dec;102(12):11067–80. doi: 10.3168/jds.2019-16645 31563317

[6] Chen C, Steibel JP, Tempelman RJ. Genome-Wide Association Analyses Based on Broadly Different Specifications for Prior Distributions, Genomic Windows, and Estimation Methods. Genetics. 2017 Aug 1;206(4):1791–806. doi: 10.1534/genetics.117.202259 28637709 PMC5560788

[7] Li J, Wang Z, Fernando R, Cheng H. Tests of association based on genomic windows can lead to spurious associations when using genotype panels with heterogeneous SNP densities. Genet Sel Evol. 2021 Dec;53(1):45. doi: 10.1186/s12711-021-00638-x 34039266 PMC8157676

[8] Aguilar I, Legarra A, Cardoso F, Masuda Y, Lourenco D, Misztal I. Frequentist p-values for large-scale-single step genome-wide association, with an application to birth weight in American Angus cattle. Genet Sel Evol. 2019 Dec;51(1):28. doi: 10.1186/s12711-019-0469-3 31221101 PMC6584984

[9] Buaban S, Lengnudum K, Boonkum W, Phakdeedindan P. Genome-wide association study on milk production and somatic cell score for Thai dairy cattle using weighted single-step approach with random regression test-day model. J Dairy Sci. 2022 Jan;105(1):468–94. doi: 10.3168/jds.2020-19826 34756438

[10] Zhou C, Li C, Cai W, Liu S, Yin H, Shi S, et al. Genome-Wide Association Study for Milk Protein Composition Traits in a Chinese Holstein Population Using a Single-Step Approach. Front Genet. 2019 Feb 19;10:72. doi: 10.3389/fgene.2019.00072 30838020 PMC6389681

[11] Zhuang Z, Xu L, Yang J, Gao H, Zhang L, Gao X, et al. Weighted Single-Step Genome-Wide Association Study for Growth Traits in Chinese Simmental Beef Cattle. Genes. 2020 Feb 11;11(2):189. doi: 10.3390/genes11020189 32053968 PMC7074168

[12] García-Ruiz A, Ruiz F de J, Van Tassell CP, Montaldo HH. Genome Regions Associated to Milk Production Traits and Somatic Cell Score in the Mexican Holstein Population. In: Proceedings, 10th World Congress of Genetics Applied to Livestock Production [Internet]. Vancouver, BC Canadá; 2014 [cited 2024 Jun 13]. Available from: https://www.wcgalp.com/

[13] Dabdoub SM, Shook GE. Phenotypic relations among milk yield, somatic cell count and clinical mastitis. J Dairy Sci. 1984;(67):163–4.

[14] Illumina. Illumina, Inc. [Internet]. [cited 2024 Jul 2]. Available from: https://support.illumina.com/downloads/bovineld-v2-0-product-files.html

[15] NEOGEN. © Neogen Corporation [Internet]. 2024. Available from: https://www.neogen.com/categories/genotyping-arrays/?q=12&s=MostPopular&c=%7CBrand;GeneSeek%C2%AE+Genomic+Profiler%E2%84%A2

[16] Axiom. Thermo Fisher Scientific Inc. [Internet]. Available from: https://www.thermofisher.com/order/catalog/product/901791

[17] García-Ruiz A, Ruiz-Lopez FJ, Wiggans GR, Van Tassell CP, Montaldo HH. Effect of reference population size and available ancestor genotypes on imputation of Mexican Holstein genotypes. J Dairy Sci. 2015 May;98(5):3478–84. doi: 10.3168/jds.2014-9132 25771055

[18] VanRaden PM, O’Connell JR, Wiggans GR, Weigel KA. Genomic evaluations with many more genotypes. Genet Sel Evol. 2011 Dec;43(1):10. doi: 10.1186/1297-9686-43-10 21366914 PMC3056758

[19] Aguilar I, Misztal I, Tsuruta S, Legarra A, Huiyu Wang. PREGSF90 –POSTGSF90: Computational Tools for the Implementation of Single-step Genomic Selection and Genome-wide Association with Ungenotyped Individuals in BLUPF90 Programs. 2014 [cited 2023 Mar 9]; Available from: http://rgdoi.net/10.13140/2.1.4801.5045

[20] Wang H, Misztal I, Aguilar I, Legarra A, Muir WM. Genome-wide association mapping including phenotypes from relatives without genotypes. Genet Res. 2012 Apr;94(2):73–83. doi: 10.1017/S0016672312000274 22624567

[21] Lourenco D, Legarra A, Tsuruta S, Masuda Y, Aguilar I, Misztal I. Single-Step Genomic Evaluations from Theory to Practice: Using SNP Chips and Sequence Data in BLUPF90. Genes. 2020 Jul 14;11(7):790. doi: 10.3390/genes11070790 32674271 PMC7397237

[22] Misztal I, Lourenco D, Aguilar I, Legarra A, Vitezica Z. Manual for BLUPF90 family of programs [Internet]. 2022. 149 p. Available from: http://nce.ads.uga.edu/wiki/lib/exe/fetch.php?media=blupf90_all8.pdf

[23] Martínez-Marín GJ, García-Ruiz A, Vásquez-Peláez CG, Román-Ponce SI, Ruiz-López FJ. Effect of calving age on genetic evaluation of milk yield in Holstein cattle. Trop Anim Health Prod. 2020 Jan;52(1):365–71. doi: 10.1007/s11250-019-02023-9 31359354

[24] Masuda Y. Introduction to BLUPF90 suite programs. 2019 Sep 1;1.0.1:189.

[25] Purcell S, Neale B, Todd-Brown K, Thomas L, Ferreira MAR, Bender D, et al. PLINK: A Tool Set for Whole-Genome Association and Population-Based Linkage Analyses. Am J Hum Genet. 2007 Sep;81(3):559–75. doi: 10.1086/519795 17701901 PMC1950838

[26] Wang H, Misztal I, Aguilar I, Legarra A, Fernando RL, Vitezica Z, et al. Genome-wide association mapping including phenotypes from relatives without genotypes in a single-step (ssGWAS) for 6-week body weight in broiler chickens. Front Genet [Internet]. 2014 May 20 [cited 2023 Mar 10];5. Available from: http://journal.frontiersin.org/article/10.3389/fgene.2014.00134/abstract 10.3389/fgene.2014.00134 PMC4033036 24904635

[27] Aguilar I, Misztal I, Johnson DL, Legarra A, Tsuruta S, Lawlor TJ. Hot topic: A unified approach to utilize phenotypic, full pedigree, and genomic information for genetic evaluation of Holstein final score. J Dairy Sci. 2010 Feb;93(2):743–52. doi: 10.3168/jds.2009-2730 20105546

[28] VanRaden PM. Efficient Methods to Compute Genomic Predictions. J Dairy Sci. 2008 Nov;91(11):4414–23. doi: 10.3168/jds.2007-0980 18946147

[29] Yang Q, Cui J, Chazaro I, Cupples LA, Demissie S. Power and type I error rate of false discovery rate approaches in genome-wide association studies. BMC Genet. 2005 Dec;6(S1):S134. doi: 10.1186/1471-2156-6-S1-S134 16451593 PMC1866802

[30] Jiang J, Ma L, Prakapenka D, Tooker ME, VanRaden PM, Cole JB, et al. Extreme antagonistic pleiotropy effects of DGAT1 on fat, milk and protein yields. In: World Congress on Genetics Applied to Livestock [Internet]. 2018. Available from: https://www.aipl.arsusda.gov/publish/other/2018/WCGALP2018_Jiang.pdf

[31] Legarra A, Lourenco DAL, Vitezica ZG. Bases for Genomic Prediction [Internet]. 2018. 141 p. Available from: http://genoweb.toulouse.inra.fr/~alegarra/GSIP.pdf

[32] Marques DBD, Bastiaansen JWM, Broekhuijse MLWJ. Weighted single-step GWAS and gene network analysis reveal new candidate genes for semen traits in pigs. Genet Sel Evol [Internet]. 2018;50(40). Available from: doi: 10.1186/s12711-018-0412-z 30081822 PMC6080523

[33] Hu ZL, Park CA, Reecy JM. Bringing the Animal QTLdb and CorrDB into the future: meeting new challenges and providing updated services. Nucleic Acids Res. 2022 Jan 7;50(D1):D956–61. doi: 10.1093/nar/gkab1116 34850103 PMC8728226

[34] Szklarczyk D, Kirsch R, Koutrouli M, Nastou K, Mehryary F, Hachilif R, et al. The STRING database in 2023: protein–protein association networks and functional enrichment analyses for any sequenced genome of interest. Nucleic Acids Res. 2023 Jan 6;51(D1):D638–46. doi: 10.1093/nar/gkac1000 36370105 PMC9825434

[35] Li C, Sun D, Zhang S, Yang S, Alim MA, Zhang Q, et al. Genetic effects of FASN, PPARGC1A, ABCG2 and IGF1 revealing the association with milk fatty acids in a Chinese Holstein cattle population based on a post genome-wide association study. BMC Genet. 2016 Dec;17(1):110. 27468856 10.1186/s12863-016-0418-x PMC4963957

[36] Cortes-Hernández JG, Ruiz-López FJ, Vásquez-Peláez CG, García-Ruiz A. Runs of homocigosity and its association with productive traits in Mexican Holstein cattle. Tomaszewska E, editor. PLOS ONE. 2022 Sep 19;17(9):e0274743. doi: 10.1371/journal.pone.0274743 36121861 PMC9484644

[37] Durán Aguilar M, Román Ponce SI, Ruiz López FJ, González Padilla E, Vásquez Peláez CG, Bagnato A, et al. Genome‐wide association study for milk somatic cell score in holstein cattle using copy number variation as markers. J Anim Breed Genet. 2017 Feb;134(1):49–59. doi: 10.1111/jbg.12238 27578198

[38] Kim S, Lim B, Cho J, Lee S, Dang CG, Jeon JH, et al. Genome-Wide Identification of Candidate Genes for Milk Production Traits in Korean Holstein Cattle. Animals. 2021 May 13;11(5):1392. doi: 10.3390/ani11051392 34068321 PMC8153329

[39] Liu L, Zhou J, Chen CJ, Zhang J, Wen W, Tian J, et al. GWAS-Based Identification of New Loci for Milk Yield, Fat, and Protein in Holstein Cattle. Animals. 2020 Nov 5;10(11):2048. doi: 10.3390/ani10112048 33167458 PMC7694478

[40] Bobbo T, Tiezzi F, Penasa M, De Marchi M, Cassandro M. Short communication: Association analysis of diacylglycerol acyltransferase (DGAT1) mutation on chromosome 14 for milk yield and composition traits, somatic cell score, and coagulation properties in Holstein bulls. J Dairy Sci. 2018 Sep;101(9):8087–91. doi: 10.3168/jds.2018-14533 30007808

[41] Atashi H, Salavati M, De Koster J, Ehrlich J, Crowe M, Opsomer G, et al. Genome‐wide association for milk production and lactation curve parameters in Holstein dairy cows. J Anim Breed Genet. 2020 May;137(3):292–304. doi: 10.1111/jbg.12442 31576624 PMC7217222

[42] Ariyarathne HBPC, Correa-Luna M, Blair HT, Garrick DJ, Lopez-Villalobos N. Identification of Genomic Regions Associated with Concentrations of Milk Fat, Protein, Urea and Efficiency of Crude Protein Utilization in Grazing Dairy Cows. Genes. 2021 Mar 23;12(3):456. doi: 10.3390/genes12030456 33806889 PMC8004844

[43] Silva AA, Silva DA, Silva FF, Costa CN, Silva HT, Lopes PS, et al. GWAS and gene networks for milk-related traits from test-day multiple lactations in Portuguese Holstein cattle. J Appl Genet. 2020 Sep;61(3):465–76. doi: 10.1007/s13353-020-00567-3 32607783

[44] Prakapenka D, Liang Z, Jiang J, Ma L, Da Y. A Large-Scale Genome-Wide Association Study of Epistasis Effects of Production Traits and Daughter Pregnancy Rate in U.S. Holstein Cattle. Genes. 2021 Jul 18;12(7):1089. doi: 10.3390/genes12071089 34356105 PMC8304971

[45] Peters SO, Kızılkaya K, Ibeagha-Awemu EM, Sinecen M, Zhao X. Comparative accuracies of genetic values predicted for economically important milk traits, genome-wide association, and linkage disequilibrium patterns of Canadian Holstein cows. J Dairy Sci. 2021 Feb;104(2):1900–16. doi: 10.3168/jds.2020-18489 33358789

[46] Schopen GCB, Visker MHPW, Koks PD, Mullaart E, Van Arendonk JAM, Bovenhuis H. Whole-genome association study for milk protein composition in dairy cattle. J Dairy Sci. 2011 Jun;94(6):3148–58. doi: 10.3168/jds.2010-4030 21605784

[47] Lu X, Jiang H, Arbab AAI, Wang B, Liu D, Abdalla IM, et al. Investigating Genetic Characteristics of Chinese Holstein Cow’s Milk Somatic Cell Score by Genetic Parameter Estimation and Genome-Wide Association. Agriculture. 2023 Jan 21;13(2):267.

[48] Guinan FL, Wiggans GR, Norman HD, Dürr JW, Cole JB, Van Tassell CP, et al. Changes in genetic trends in US dairy cattle since the implementation of genomic selection. J Dairy Sci. 2023 Feb;106(2):1110–29. doi: 10.3168/jds.2022-22205 36494224

