# Supplementary material for: Uncovering the genetic basis of milk production traits in Mexican Holstein cattle based on individual markers and genomic windows
Source: PLoS One. 2025 Feb 3;20(2):e0314888. doi: 10.1371/journal.pone.0314888 (PMC11790082; doi:10.1371/journal.pone.0314888)
Supplement: S1 Table — ALB: Blood albumin level, UH: Udder height, SNPn: SNP name, BH: β-Hydroxybutyrate content in milk, BTA: Bos taurus autosome, BTW: Birth weight, BW: Body weight, CA: Caprylic acid content in milk, CAL: Calcium content in milk, CAS: Casein content in milk, CE: Calving ease, CO: Milk cholesterol content, CR: Conception rate, CSW: Carcass weight, DMI: Dry matter intake, EV: Ejaculate volume, FC: Fat content, FE: Milk iron content, FX: Fertility index, FY: Fat yield, HI: Height, HT: Heat tolerance, KP: Milk kappa casein content, KT: Ketosis, LAC: Milk lactose production, LI: Linoleic acid content in milk, LIN: Linolenic acid content in milk, LM: Lean meat yield, LON: Longevity, LPL: Length of productive life, MB: Position in Mb, MI: Myristic acid content in milk, MS: Milking speed, MY: Milk yield, NM: Net merit, SB: Stillbirth, OL: Oleic acid content in milk, PAL: Palmitic acid content in milk, PAT: Palmitoleic acid content in milk, PC: Protein content, PLAC: Persistence of lactation, PY: Protein yield, P-FC: p-value for association of fat content, P-FY: p-value for association of fat yield, P-MY: p-value for association of milk yield, P-PC: p-value for association of protein content, P-PY: p-value for association of protein yield, QTL: Quantitative trait locus, RVL: Rear view of hind legs, RIV: Riboflavin content in milk, SCS: Somatic cell score, TP: Teat placement, TS: Tuberculosis susceptibility, UCF: Udder conformation, UC: Udder cleft. ASSOCIATIONS, GENES, and QTLs were searched on the QTLdb website https://www.animalgenome.org/cgi-bin/QTLdb/index [34] with the SNP location used as a reference. (DOCX) [file pone.0314888.s001.docx]

| SNPn | BTA | MB | P-MY | P-FY | P-FP | P-PY | P-PP | ASSOCIATIONS | GENES | QTL |
| --- | --- | --- | --- | --- | --- | --- | --- | --- | --- | --- |
| ARS-BFGL-NGS-100206 | 1 | 133.39 |  | 7.04 |  |  |  | BTW and EV. |  |  |
| ARS-BFGL-NGS-100480 | 14 | 3.34 |  | 9.54 | 15.39 |  |  | PY and FY. |  |  |
| ARS-BFGL-NGS-101653 | 14 | 1.12 |  | 10.11 | 19.30 |  |  | MY, PY and FY. |  |  |
| ARS-BFGL-NGS-103064 | 14 | 1.62 |  | 7.11 | 21.57 |  |  | FY, PY and MY. | CYP11B. |  |
| ARS-BFGL-NGS-110176 | 22 | 16.68 |  | 8.29 |  | 7.58 |  | LON. |  |  |
| ARS-BFGL-NGS-112812 | 6 | 37.19 |  |  |  |  | 7.15 | BW and PP. |  |  |
| ARS-BFGL-NGS-115947 | 14 | 4.46 |  |  | 12.49 |  |  | FY and PY. |  |  |
| ARS-BFGL-NGS-118998 | 20 | 32.01 |  |  |  |  | 6.98 | FP, PP and MY. | GHR. |  |
| ARS-BFGL-NGS-13586 | 3 | 15.33 |  |  |  |  | 7.77 | PP. |  |  |
| ARS-BFGL-NGS-18365 | 14 | 0.92 |  | 8.20 | 32.97 |  |  | MY, KT, FY, PY and CO. |  |  |
| ARS-BFGL-NGS-22111 | 14 | 3.07 |  |  | 8.35 |  |  | FY, PY, LI, PAT and OL. |  |  |
| ARS-BFGL-NGS-22866 | 14 | 1.68 |  | 7.19 | 8.65 |  |  | FY and PY. |  |  |
| ARS-BFGL-NGS-26520 | 14 | 1.18 |  | 12.29 | 25.94 |  |  | FP, PP, LI, CA, PAT and OL. | ZC3H3. |  |
| ARS-BFGL-NGS-3122 | 14 | 1.52 |  |  | 13.13 |  |  | MY. | GML. |  |
| ARS-BFGL-NGS-34135 | 14 | 0.49 |  | 17.52 | 79.83 |  | 16.20 | MY, FY, PY, OL, LI, LIN, PAL and MI. |  |  |
| ARS-BFGL-NGS-35038 | 16 | 48.29 |  | 7.44 |  |  |  |  |  |  |
| ARS-BFGL-NGS-4939 | 14 | 0.61 | 16.04 | 37.71 | 230.54 | 10.49 | 47.64 | FY and MY. | DGAT1 and EXOSC4. | FP and PY. |
| ARS-BFGL-NGS-56327 | 14 | 3.31 |  | 7.02 |  |  |  | FY, MY, PY, SCS and RVL. |  |  |
| ARS-BFGL-NGS-57820 | 14 | 0.47 | 14.52 | 31.27 | 192.52 | 10.12 | 32.94 | MY, PP, PY, FY, FP, RIV, PAT and LIN. | PPP1R16A and FOXH1. |  |
| ARS-BFGL-NGS-59769 | 14 | 1.85 |  |  | 21.91 |  |  | MY, FY and PY. | ADGRB1. |  |
| ARS-BFGL-NGS-64215 | 3 | 15.47 |  |  |  |  | 14.74 | MY, FP and PP. | EFNA1. |  |
| ARS-BFGL-NGS-71749 | 14 | 0.76 |  | 7.44 | 23.81 |  |  | FP, PP and MY. | EXOSC4 and OPLAH. |  |
| ARS-BFGL-NGS-84473 | 10 | 59.37 | 7.22 |  |  |  |  | MS. |  |  |
| ARS-BFGL-NGS-85419 | 14 | 2.09 |  |  | 7.70 |  |  | MY, FP, PP, LI, LIN, OL, PAL and PAT. |  |  |
| ARS-BFGL-NGS-94706 | 14 | 0.51 |  | 16.50 | 75.94 |  | 15.29 | FP, PP, MY and UCF. |  |  |
| BTA-121739-no-rs | 6 | 36.64 |  |  | 10.57 |  | 25.48 | PP. | PYD2. |  |
| BTA-34956-no-rs | 14 | 0.33 |  |  | 30.15 |  | 8.30 | MY, FP and PP. |  |  |
| BTA-35941-no-rs | 14 | 1.08 |  | 12.78 | 34.12 |  | 10.67 | BH, FP, PP, MY, LI, MI, PAL, PAT and CO. |  |  |
| BTB-01023946 | 29 | 34.56 | 9.86 |  |  |  |  |  |  |  |
| BTB-02095583 | 16 | 48.33 |  | 8.66 |  | 7.13 |  |  |  |  |
| BovineHD0100034107 | 1 | 119.53 |  | 8.97 |  | 7.17 |  |  |  |  |
| BovineHD0300005051 | 3 | 15.39 |  |  |  |  | 12.81 | FP and PP. |  |  |
| BovineHD0300005253 | 3 | 15.96 |  |  |  |  | 8.53 | PP. |  |  |
| BovineHD0500024736 | 5 | 86.82 |  | 7.34 |  |  |  |  |  |  |
| BovineHD0500024796 | 5 | 87.03 |  | 8.07 |  |  |  | FP. |  |  |
| BovineHD0500025193 | 5 | 88.43 |  | 8.18 |  |  |  | FP and CR. |  |  |
| BovineHD0500025605 | 5 | 89.74 |  | 7.63 |  |  |  | FP. |  |  |
| BovineHD0500026249 | 5 | 92.03 |  |  | 8.03 |  |  | FP. |  |  |
| BovineHD0500026635 | 5 | 93.41 |  |  | 9.52 |  |  | FP and PAL. |  |  |
| BovineHD0500026655 | 5 | 93.50 |  | 7.57 | 16.56 |  |  | FP, MY and PAL. | MGST1. |  |
| BovineHD0500026662 | 5 | 93.52 |  | 13.04 | 28.80 |  |  | FP. |  |  |
| BovineHD0500026682 | 5 | 93.57 |  | 13.68 | 16.88 |  |  | FP. | SLC15A5. |  |
| BovineHD0500026737 | 5 | 93.72 |  |  | 7.16 |  |  | MY, FP, PP and KP. |  |  |
| BovineHD0500026872 | 5 | 94.19 |  |  | 11.81 |  |  | FP and FY. | EPS8. |  |
| BovineHD0500027282 | 5 | 95.70 |  | 7.96 |  |  |  | FP and FY. |  |  |
| BovineHD0600006457 | 6 | 22.07 | 6.97 |  |  |  |  |  |  |  |
| BovineHD0600010422 | 6 | 36.20 |  |  |  |  | 7.37 | PP. |  |  |
| BovineHD0600010427 | 6 | 36.25 |  |  |  |  | 8.99 | DMI. |  |  |
| BovineHD0600010429 | 6 | 36.26 |  |  |  |  | 7.96 |  |  |  |
| BovineHD0600010430 | 6 | 36.26 |  |  |  |  | 8.06 |  |  |  |
| BovineHD0600010435 | 6 | 36.30 |  |  |  |  | 9.37 | LAC, FP and PY. | ABCG2. |  |
| BovineHD0600010480 | 6 | 36.44 |  |  |  |  | 6.99 |  |  |  |
| BovineHD0600010481 | 6 | 36.44 |  |  |  |  | 7.23 |  |  |  |
| BovineHD0600010552 | 6 | 36.58 |  |  |  |  | 10.28 |  |  |  |
| BovineHD0600010555 | 6 | 36.59 |  |  | 11.61 |  | 26.66 | LAC and PP. | ABCG2. |  |
| BovineHD0600010569 | 6 | 36.66 |  |  | 10.88 |  | 26.58 | LAC, PP and FP. | ABCG2. |  |
| BovineHD0600010574 | 6 | 36.68 |  |  |  |  | 12.34 |  |  |  |
| BovineHD0600010576 | 6 | 36.69 |  |  |  |  | 10.71 | MY. |  |  |
| BovineHD0600010605 | 6 | 36.86 |  |  |  |  | 10.63 | FP and PP. | ABCG2. |  |
| BovineHD0600010606 | 6 | 36.86 |  |  |  |  | 10.48 |  |  |  |
| BovineHD0600010624 | 6 | 36.94 |  |  | 7.21 |  | 17.59 | PP. |  |  |
| BovineHD0600010625 | 6 | 36.94 |  |  | 8.31 |  | 21.45 | PP. |  |  |
| BovineHD0600010630 | 6 | 36.97 |  |  | 13.35 |  | 38.91 | FP and PP. |  |  |
| BovineHD0600010908 | 6 | 38.33 |  |  |  |  | 7.85 |  |  |  |
| BovineHD0600010909 | 6 | 38.33 |  |  |  |  | 7.86 |  |  |  |
| BovineHD0600010912 | 6 | 38.35 |  |  |  |  | 7.85 |  |  |  |
| BovineHD0600010922 | 6 | 38.41 |  |  |  |  | 6.97 |  |  |  |
| BovineHD0600010931 | 6 | 38.47 |  |  |  |  | 11.95 |  |  |  |
| BovineHD0600010932 | 6 | 38.47 |  |  |  |  | 7.96 | PP. |  |  |
| BovineHD0600010933 | 6 | 38.47 |  |  |  |  | 8.10 | PP. |  |  |
| BovineHD0600010934 | 6 | 38.47 |  |  |  |  | 12.14 | PP. |  |  |
| BovineHD0600010936 | 6 | 38.49 |  |  |  |  | 11.82 | PP. |  |  |
| BovineHD0600023906 | 6 | 85.62 |  |  |  |  | 7.47 | PP and KP. |  |  |
| BovineHD0600023926 | 6 | 85.69 |  |  |  |  | 7.15 | PP Y KP, MY and SCS. |  |  |
| BovineHD0600023965 | 6 | 85.84 |  |  |  |  | 8.74 | PY, PP, SCS and KP. |  |  |
| BovineHD1000017198 | 10 | 58.11 | 7.46 |  |  |  |  | MS and HI. |  |  |
| BovineHD1000017422 | 10 | 59.21 | 7.16 |  |  |  |  | MS and TS. |  |  |
| BovineHD1400000143 | 14 | 0.24 |  |  | 35.87 |  | 8.80 | FP. | ZNF16. |  |
| BovineHD1400000152 | 14 | 0.26 |  |  | 38.28 |  | 8.27 | FY, PY, FP, PP and MY. |  |  |
| BovineHD1400000187 | 14 | 0.40 |  |  | 25.92 |  |  | FY, PY, FP, PP, MY, MI, PAL and PAT. |  |  |
| BovineHD1400000243 | 14 | 0.68 | 7.13 | 10.36 | 74.18 |  | 13.77 | PP. | MROH1. | PP. |
| BovineHD1400000246 | 14 | 0.69 | 7.51 | 11.12 | 80.76 |  | 14.85 | FP and PP. | MROH1. |  |
| BovineHD1400000249 | 14 | 0.70 | 7.62 | 10.85 | 76.76 |  | 15.10 | FP and PP. | MROH1. |  |
| BovineHD1400000262 | 14 | 0.78 | 12.75 | 23.71 | 121.68 |  | 23.47 | FP, PP and MY. | OPLAH. |  |
| BovineHD1400000288 | 14 | 0.89 |  | 13.75 | 35.73 |  | 9.16 | FP, PP, MY, PAT, PAL, LI and OL. |  |  |
| BovineHD1400000301 | 14 | 0.96 |  |  | 41.87 |  |  | FP, PP, MY, PAT, PAL, LI and OL. | NRBP2. |  |
| BovineHD1400000305 | 14 | 0.97 | 7.31 |  | 41.84 |  |  | FP, PP, MY, PAT, PAL, LI and OL. | PUF60 and NRBP2. |  |
| BovineHD1400000401 | 14 | 1.37 |  |  | 12.01 |  |  | FY, PY, FP and PP. |  |  |
| BovineHD1400000420 | 14 | 1.42 |  |  | 10.73 |  |  | FP, PP Y MY, PAT, PAL, LI and OL. |  |  |
| BovineHD1400000434 | 14 | 1.51 |  |  | 12.62 |  |  | KT, FY, PY, FP, PP, PAT and PAL. | LOC787628. |  |
| BovineHD1400000447 | 14 | 1.61 |  | 7.22 | 20.94 |  |  | FY, FP, PY, PP, MY, OL, LI, LIN, PAL and CA. | LY6K. |  |
| BovineHD1400000453 | 14 | 1.66 |  | 9.35 | 18.89 |  |  | FP, PP, MY, PAT, LI and OL. | LY6D. |  |
| BovineHD1400000476 | 14 | 1.77 |  |  | 10.16 |  |  | MY, PY, FP, PP and PAT. |  |  |
| BovineHD1400000479 | 14 | 1.78 |  |  | 13.73 |  |  | MY, PY, FP, PP, PAT, PAL, PAT and OL. | ADGRB1. |  |
| BovineHD1400000616 | 14 | 2.22 |  | 9.79 | 29.80 |  | 8.23 | MY, PP, PY, FY, FP, RIV, PAT, PAL, LI and LIN. | TSNARE1. |  |
| BovineHD1400000788 | 14 | 2.84 |  |  | 9.50 |  |  | MY, PP, PY, FY, FP, MI, FE, LI, LIN and OL. | PTK2. |  |
| BovineHD1400000809 | 14 | 2.91 |  |  | 9.35 |  |  | FY, FP, LI, LIN, PAT, PAL and CA. |  |  |
| BovineHD1400000851 | 14 | 3.06 |  |  | 8.43 |  |  | MY, PP, FY, FP, LIN, LI, PAT, PAL and OL. |  |  |
| BovineHD1400000977 | 14 | 3.39 |  |  | 14.96 |  |  | MY, FY, FP, PY, PP, OL, LI, PAT and PAL. | TRAPCC9. |  |
| BovineHD1400000999 | 14 | 3.43 |  |  | 11.36 |  |  | MY, FY, FP, PY, PP, OL, LI, LIN, PAT and PAL. | TRAPCC9. |  |
| BovineHD1400016503 | 14 | 57.35 | 7.49 |  |  |  |  |  |  |  |
| BovineHD1400016730 | 14 | 58.15 | 7.27 |  |  |  |  |  |  |  |
| BovineHD1400018109 | 14 | 62.77 | 7.39 |  |  |  |  |  |  |  |
| BovineHD1400018541 | 14 | 64.08 |  |  |  |  | 16.22 | BH, KT, PP and FP. | RNF19A. |  |
| BovineHD1400018544 | 14 | 64.10 |  |  |  |  | 14.89 | FP and PP. |  |  |
| BovineHD1400018551 | 14 | 64.13 |  |  |  |  | 16.09 | FP and PP. |  |  |
| BovineHD1400018576 | 14 | 64.25 |  |  |  |  | 13.01 | KT, PP and FP. |  |  |
| BovineHD1400018582 | 14 | 64.27 |  |  |  |  | 16.35 | KT, PP, FP and MY. | RGS22. |  |
| BovineHD1400018987 | 14 | 65.80 |  |  |  |  | 10.49 | PP, FP and MY. | STK3. |  |
| BovineHD1800004933 | 18 | 15.64 |  | 7.15 |  |  |  |  |  |  |
| BovineHD2000001599 | 20 | 5.14 | 7.53 |  |  |  |  |  |  |  |
| BovineHD2000001600 | 20 | 5.14 | 7.48 |  |  |  |  |  |  |  |
| BovineHD2000001732 | 20 | 5.69 | 7.23 |  |  | 7.04 |  |  |  |  |
| BovineHD2000009226 | 20 | 32.03 |  |  |  |  | 10.05 | MY, FP, PP and PLAC. | GHR. |  |
| BovineHD2000009251 | 20 | 32.08 |  |  |  |  | 7.84 | PP, FP and PLAC. |  |  |
| BovineHD2000009307 | 20 | 32.37 |  |  |  |  | 7.86 | MY, FP and PP. | GHR. |  |
| BovineHD4100004496 | 6 | 36.13 |  |  |  |  | 7.84 | FP and PP. | HERC3. |  |
| BovineHD4100004501 | 6 | 36.17 |  |  |  |  | 8.20 |  |  |  |
| BovineHD4100004545 | 6 | 36.86 |  |  |  |  | 9.74 |  |  |  |
| BovineHD4100004546 | 6 | 36.86 |  |  |  |  | 9.51 |  |  |  |
| BovineHD4100004547 | 6 | 36.87 |  |  |  |  | 10.61 |  |  |  |
| BovineHD4100004557 | 6 | 36.94 |  |  | 8.08 |  | 21.35 |  |  |  |
| BovineHD4100004558 | 6 | 36.96 |  |  | 12.19 |  | 37.43 | PP and FP. |  |  |
| BovineHD4100004560 | 6 | 36.97 |  |  | 10.44 |  | 27.23 | PP and FP. |  |  |
| BovineHD4100004580 | 6 | 37.42 |  |  |  |  | 7.56 |  |  |  |
| BovineHD4100004586 | 6 | 37.52 | 8.88 |  |  |  | 9.21 | HI. |  |  |
| BovineHD4100004675 | 6 | 38.32 |  |  |  |  | 7.44 |  |  |  |
| BovineHD4100004679 | 6 | 38.35 |  |  |  |  | 7.81 |  |  |  |
| BovineHD4100010534 | 14 | 1.20 |  | 13.94 | 31.24 |  | 10.19 | CE, CAL, PP, PY, FP, FY, PAT, PAL, OL, LIN, MI and LI. | ZC3H3. |  |
| BovineHD4100010542 | 14 | 1.29 |  |  | 7.13 |  |  | PY, FY, PP, FP, OL, PAT and PAL. |  |  |
| Hapmap24715-BTC-001973 | 14 | 1.04 |  | 9.10 | 14.44 |  |  | MY, FY, FP, PY and PP. |  |  |
| Hapmap24717-BTC-002824 | 14 | 1.80 |  |  | 9.67 |  |  | MY, FY, FP, PY, PP, CAL and MS. |  |  |
| Hapmap25384-BTC-001997 | 14 | 1.02 |  | 9.89 | 16.78 |  |  | MY, FY, FP, PY and PP. | IQANK1. |  |
| Hapmap26259-BTC-033526 | 6 | 36.89 |  |  |  |  | 7.70 | BW. |  |  |
| Hapmap26264-BTC-037159 | 6 | 36.16 |  |  |  |  | 8.49 | BW. |  |  |
| Hapmap26527-BTC-005059 | 14 | 3.15 |  |  | 7.27 |  |  | MY, FY, FP, PY and PP. | CHRAC1. |  |
| Hapmap27298-BTC-035654 | 6 | 38.33 |  |  |  |  | 13.37 |  |  |  |
| Hapmap27703-BTC-053907 | 14 | 3.55 |  |  | 11.49 |  |  | MY, FY, FP, PY and PP. | NIBP. |  |
| Hapmap29922-BTC-033565 | 6 | 36.86 |  |  |  |  | 10.08 | BW. |  |  |
| Hapmap30086-BTC-002066 | 14 | 1.32 |  | 15.16 | 45.60 |  | 11.04 | MY, FY, FP, PY, PP, MS, CAL and RIV. | GLI4. |  |
| Hapmap30374-BTC-002159 | 14 | 1.27 |  | 11.12 | 33.22 |  | 13.05 | MY, FY, FP, PY, PP, MS, CAL, CO, CAS and RIV. | RHPN1. |  |
| Hapmap30381-BTC-005750 | 14 | 0.28 |  | 12.93 | 38.55 |  | 11.88 | MY, FY, FP, PY, PP and ALB. |  |  |
| Hapmap30383-BTC-005848 | 14 | 0.31 | 7.28 | 11.90 | 73.90 |  | 15.02 | MY, HT, RIV, CO, MY, FY, FP, PY and PP. | C14H8orf33 and ZNF34. |  |
| Hapmap30646-BTC-002054 | 14 | 1.35 |  | 13.06 | 38.04 |  | 8.95 | MY, FY, FP, PY and PP. | C14H8orf33. |  |
| Hapmap30922-BTC-002021 | 14 | 0.95 |  | 7.99 | 26.22 |  |  | MY, FY, FP, PY, LM and PP. |  |  |
| Hapmap33079-BTA-163567 | 6 | 39.18 |  |  |  |  | 7.69 | BW and CSW. |  |  |
| Hapmap34051-BES7_Contig165_112 | 20 | 5.14 | 7.87 |  |  |  |  | MS. |  |  |
| Hapmap36620-SCAFFOLD50018_7571 | 14 | 2.14 |  | 7.85 | 25.33 |  | 7.66 | MY, FY, FP, PY, PP, FE, OL, LIN, LI, CO and PAT. |  |  |
| Hapmap42977-BTA-55653 | 16 | 1.97 |  |  |  |  | 9.78 | HI, TP, UH, PB, CE, UC and SB. |  |  |
| Hapmap52798-ss46526455 | 14 | 0.73 |  | 7.84 | 51.50 |  | 9.14 | LM, MY, FY, FP, PY, PP, PAL, PAT, MI and OL. | MAF1. |  |
| Hapmap54633-rs29021971 | 29 | 24.12 |  | 7.66 |  |  |  |  |  |  |
| Hapmap57625-rs29027071 | 6 | 39.80 |  |  |  |  | 9.58 |  |  |  |
| Hapmap58345-rs29010310 | 12 | 34.66 |  | 8.27 |  | 7.13 |  | FX and PLAC. |  |  |
| MS-rs109570900 | 6 | 38.78 |  |  |  |  | 7.38 |  |  |  |
| UA-IFASA-5306 | 14 | 3.44 |  |  | 8.15 |  |  | RVL, FY, NM, FP, PY, PP, OL, PAL, MI and LI. |  |  |
| UA-IFASA-6329 | 14 | 4.06 |  |  | 11.52 |  |  | MY, FY, FP, PY, PP, LI and PAT. | COL22A1. |  |
| UA-IFASA-6878 | 14 | 0.81 | 7.45 | 7.35 | 59.82 |  | 8.59 | MY, KT, CAS, TS, CO, FY, FP, PY and PP. | SPATC1 and GRINA. |  |
| UA-IFASA-7069 | 20 | 31.91 |  |  |  |  | 10.71 | LPL, PLAC, FP and PP. |  |  |
| UA-IFASA-7269 | 14 | 3.10 |  |  | 8.51 |  |  | MY, FY, FP, PP, PY, OL, LI, LIN and RIV. | EIF2C2. |  |
| UA-IFASA-7664 | 14 | 64.30 |  |  |  |  | 10.10 | MS, PP, FP and PY. | RGS22. |  |
| UA-IFASA-8997 | 14 | 1.00 | 7.14 |  | 29.03 |  |  | MY, FP, FY, PP, PY, PAT and OL. | SCRIB. |  |
| UA-IFASA-9288 | 14 | 2.93 |  |  | 7.95 |  |  | SCS, NM, RVL, LPL, FP, MY, FY, CE, PP and PY. |  |  |
|  | | | | | | | | | | |
