# Supplementary figures and images for: Uncovering the genetic basis of milk production traits in Mexican Holstein cattle based on individual markers and genomic windows
Source: PLoS One. 2025 Feb 3;20(2):e0314888. doi: 10.1371/journal.pone.0314888 (PMC11790082; doi:10.1371/journal.pone.0314888)

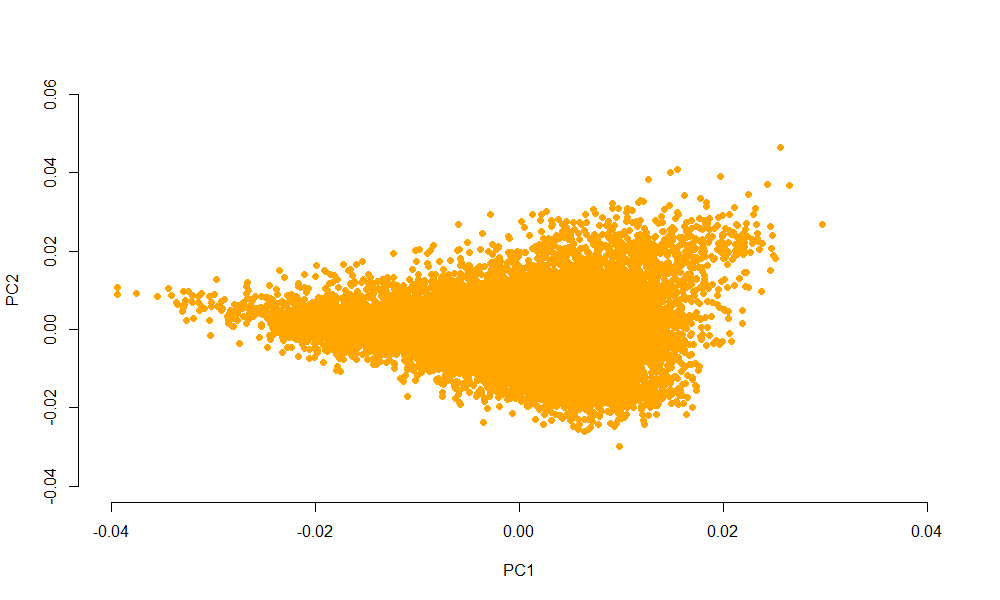

Supplement: S1 Fig — PC1: First principal component, PC2: Second principal component. (TIF) [file pone.0314888.s002.tif]

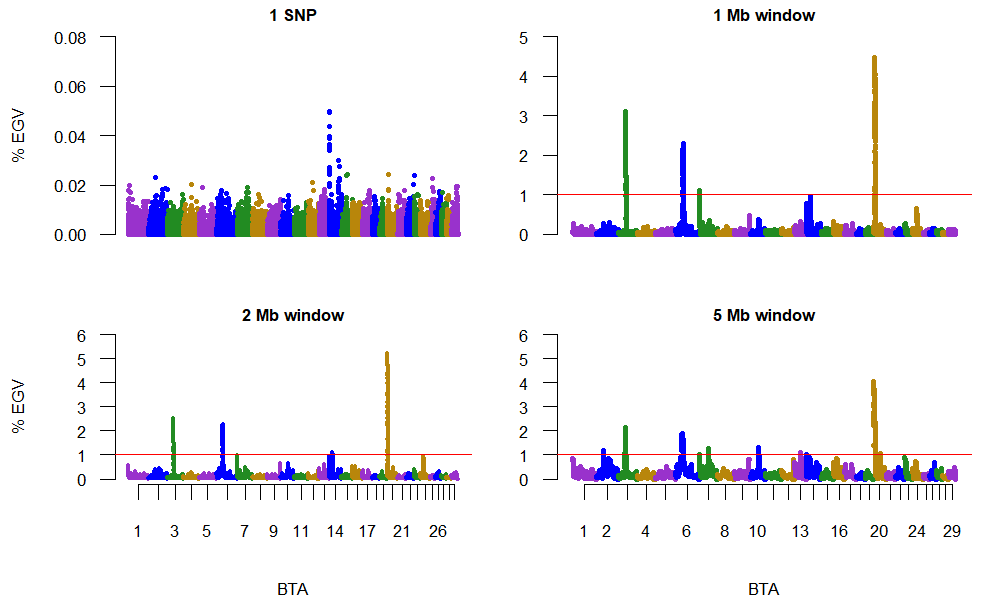

Supplement: S2 Fig — Mb: Megabase, BTA: Bos taurus autosome, MY: Milk yield, SNP: Single nucleotide polymorphism, % EGV: Explained genetic variance as a percentage. (TIF) [file pone.0314888.s003.tif]

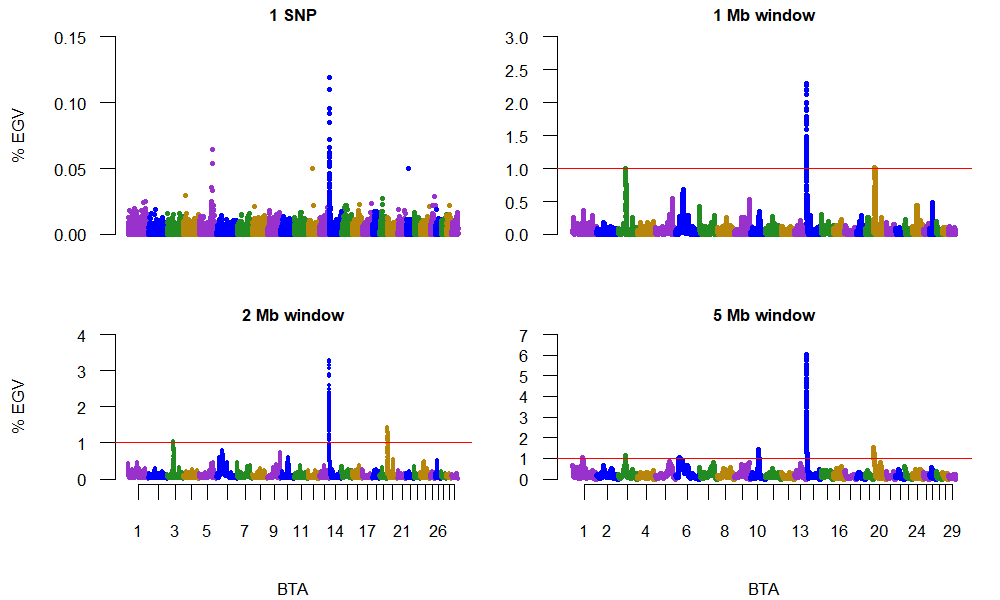

Supplement: S3 Fig — Mb: Megabase, BTA: Bos taurus autosome, FY: Fat yield, SNP: Single nucleotide polymorphism, % EGV: Explained genetic variance as percentage. (TIF) [file pone.0314888.s004.tif]

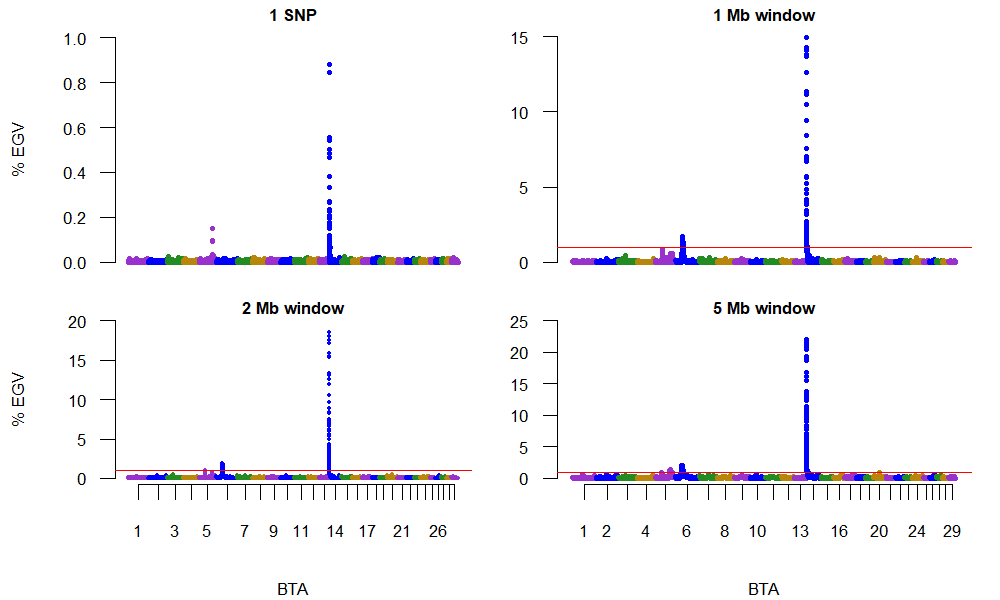

Supplement: S4 Fig — Mb: Megabase, BTA: Bos taurus autosome, FC: Fat content, SNP: Single nucleotide polymorphism, % EGV: Explained genetic variance as percentage. (TIF) [file pone.0314888.s005.tif]

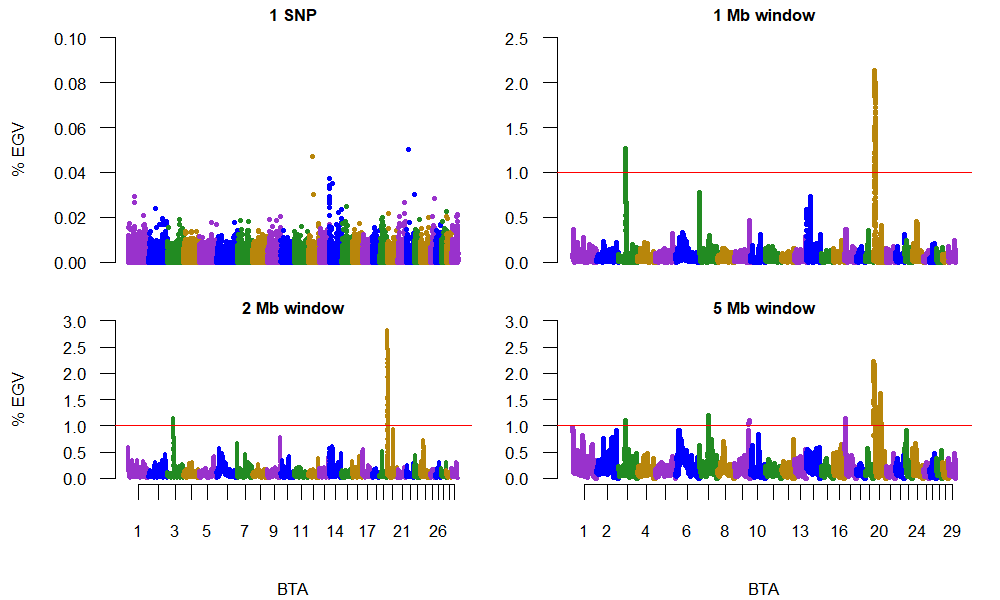

Supplement: S5 Fig — Mb: Megabase, BTA: Bos taurus autosome, PY: Protein yield, SNP: Single nucleotide polymorphism, % EGV: Explained genetic variance as percentage. (TIF) [file pone.0314888.s006.tif]

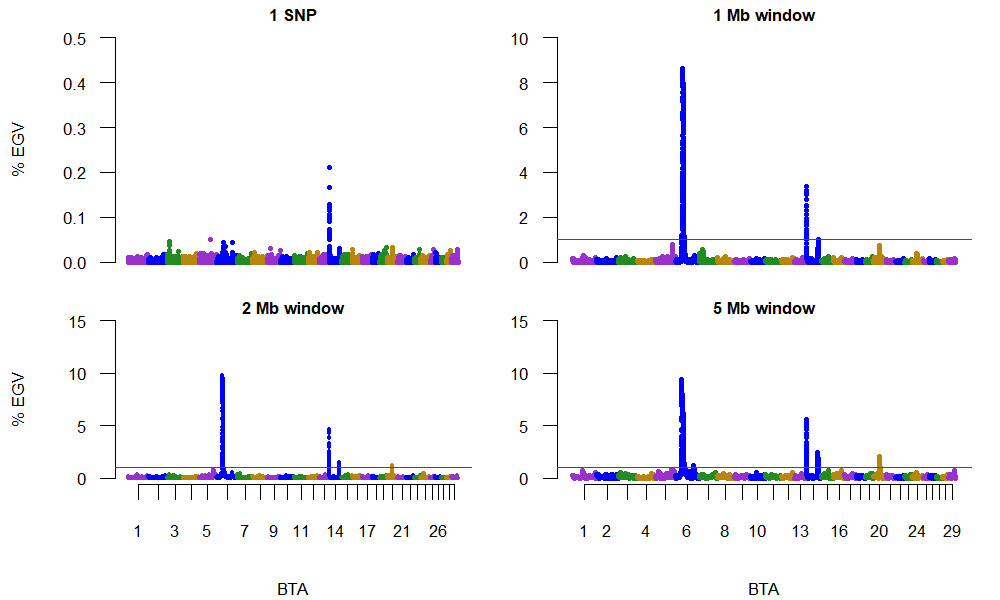

Supplement: S6 Fig — Mb: Megabase, BTA: Bos taurus autosome, PC: Protein content, SNP: Single nucleotide polymorphism, % EGV: Explained genetic variance as percentage. (TIF) [file pone.0314888.s007.tif]

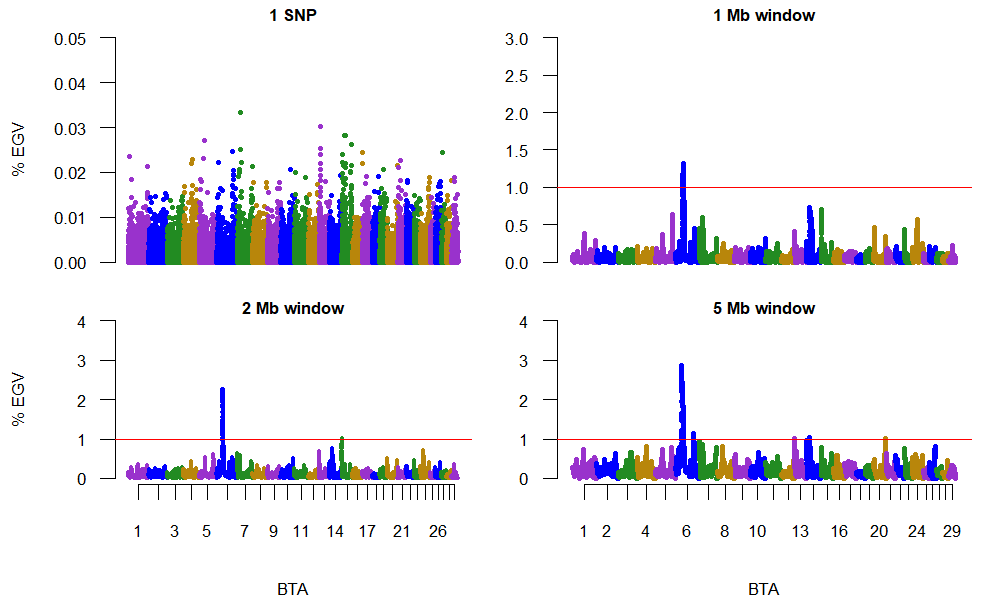

Supplement: S7 Fig — Mb: Megabase, BTA: Bos taurus autosome, SCS: Somatic cell score, SNP: Single nucleotide polymorphism, % EGV: Explained genetic variance as percentage. (TIF) [file pone.0314888.s008.tif]
